# *Caenorhabditis elegans* for research on cancer hallmarks

**DOI:** 10.1242/dmm.050079

**Published:** 2023-06-06

**Authors:** Julián Cerón

**Affiliations:** Modeling Human Diseases in C. elegans Group – Genes, Disease and Therapy Program, Bellvitge Biomedical Research Institute – IDIBELL, 08908 L'Hospitalet de Llobregat, Barcelona, Spain

**Keywords:** *Caenorhabditis elegans*, Cancer, Mutations, Tumors

## Abstract

After decades of research, our knowledge of the complexity of cancer mechanisms, elegantly summarized as ‘hallmarks of cancer’, is expanding, as are the therapeutic opportunities that this knowledge brings. However, cancer still needs intense research to diminish its tremendous impact. In this context, the use of simple model organisms such as *Caenorhabditis elegans*, in which the genetics of the apoptotic pathway was discovered, can facilitate the investigation of several cancer hallmarks. Amenable for genetic and drug screens, convenient for fast and efficient genome editing, and aligned with the 3Rs (‘Replacement, Reduction and Refinement’) principles for ethical animal research, *C. elegans* plays a significant role in unravelling the intricate network of cancer mechanisms and presents a promising option in clinical diagnosis and drug discovery.

## Teaser

**Summary:** This Review summarizes and promotes the use of *Caenorhabditis elegans* to investigate ten of the 14 cancer hallmarks that define the mechanisms of cancer.

## Introduction

**Fig. 1.**
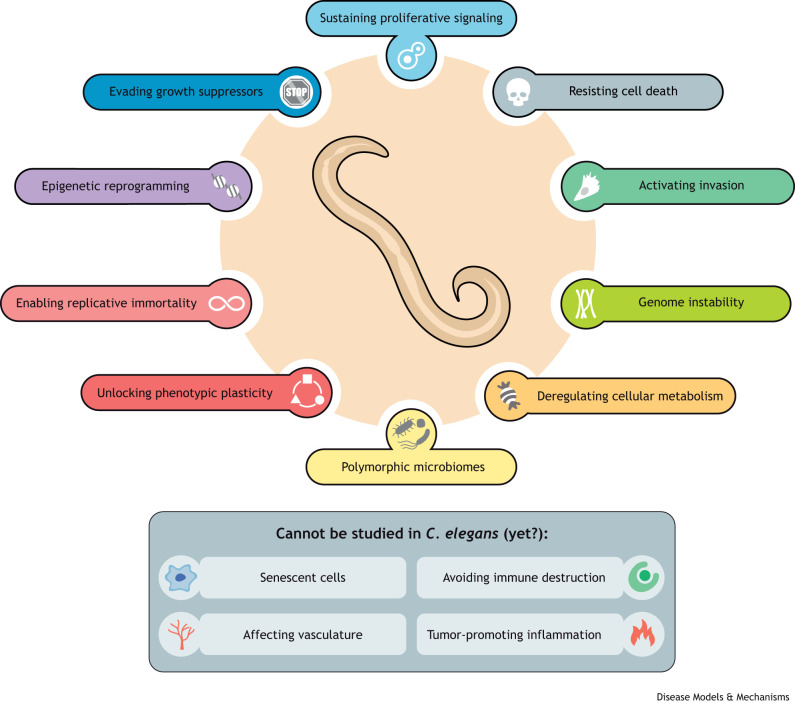
**Hallmarks of cancer that can be investigated in *C. elegans.*** Despite its relative simplicity, *C. elegans* is amenable to effective modeling of ten of the 14 hallmarks of cancer, leaving just four hallmarks that cannot (yet) be studied in this model.

Cancer remains a leading cause of morbidity and mortality worldwide, demanding significant continuous efforts from the basic, translational and clinical research communities. The ‘Hallmarks of cancer’ reviews by Hanahan and Weinberg (2000, 2011) and Hanahan (2022) have been seminal in providing conceptual clarity and direction on the tremendous complexity of cancer. To understand this complexity, the research community relies on a combination of patient data and model systems, spanning from *in vitro* platforms to mammals. Although not necessarily the obvious choice as a model organism for cancer research, the nematode worm *Caenorhabditis elegans* offers several advantages. Indeed, of the 14 hallmarks of cancer defined in the 2022 update (Hanahan, 2022), ten can be studied in *C. elegans* (Fig. 1).

*C. elegans* is a small nematode with stereotyped lineages. Hermaphrodites produce exactly 959 somatic cells. The invariant cell linage during *C. elegans* development (Box 1) provides a convenient template for studying deregulations in the complex networks controlling the balance between cell proliferation, death and differentiation. However, the lack of cell divisions in somatic adult cells restricts the studies on proliferative signaling to developing *C. elegans* larvae. Although *C. elegans* can display hyperproliferative phenotypes, they do not form malignant tumors as those seen in *Drosophila* (Gonzalez, 2013). Nevertheless, these nematodes are an effective model organism to study mechanisms leading to tumor progression. As an example, the genetics of apoptosis, which is widely dysregulated in cancer and essentially conserved from nematodes to mammals, was discovered in *C. elegans* by Bob Horvitz and earned him the Nobel Prize in Medicine or Physiology, shared with Sydney Brenner and John Sulston, in 2002.

**Box 1. *C. elegans* development and proliferative cells**

*C. elegans* is a small nematode, the body of which reaches ∼1 mm in length in adulthood. The small size, coupled with growth at atmospheric levels of oxygen and 15-25°C, and a broad availability of excellent genetic and cell biology tools, make it a popular model organism for studying a wide array of biological processes.

The embryonic development of *C. elegans* can be roughly separated into two stages: proliferation and morphogenesis. In hermaphrodites, embryonic cell divisions produce 671 cells, of which 113 undergo programmed cell death, 53 remain as blast cells and the rest terminally differentiate. After hatching, these 53 blast cells further proliferate to complete the postembryonic development that spans four tightly regulated and well-characterized larval stages, and ultimately forms an adult hermaphrodite with 959 somatic nuclei (Sulston and Horvitz, 1977; Sulston et al., 1983). Embryonic development is initially driven by transcripts obtained from the mother (maternal product) and is later completed by zygotic transcription. Postembryonic blast cell divisions begin 3 h after hatching, but only if food is available (Baugh and Sternberg, 2006). Therefore, this food dependence provides an excellent context to investigate proliferative signals from metabolic pathways. Whereas other terminally differentiated postembryonic cells are refractory to proliferative phenotypes, postembryonic blast cells (intestinal cells, P cells, seam cells and sex myoblasts) are prompt to hyperproliferate upon manipulation of some gene activities (Boxem and Van den Heuvel, 2001; Wildwater et al., 2011; Ruijtenberg and Van Den Heuvel, 2015).

Despite its status as a suitable and well-powered model organism (Table 1), a key requirement for embracing *C. elegans* as a model for cancer is to establish whether cancer genes are conserved from humans to nematodes. According to the Cancer Gene Census, more than 1% of all human genes are implicated via mutation in cancer (Tate et al., 2019). Approximately 90% of these genes bear somatic mutations and 20% germline mutations across the cancers surveyed in the Catalogue Of Somatic Mutations In Cancer (COSMIC) (Sondka et al., 2018). About half of the genes mutated in germline tumors also bear recurrent somatic mutations in sporadic tumors. To estimate how many human cancer driver genes are conserved in *C. elegans*, I took a list of 568 driver genes from the IntOgene list (Martínez-Jiménez et al., 2020), which was compiled from the analysis of 28,000 tumors of distinct cancer types, and then searched for their orthologs in the Ortholist database (Kim et al., 2018). This survey found that 72% of the human cancer driver genes have one or more orthologs in *C. elegans* (Table S1). Still, this percentage may underestimate the number of orthologs, because, for example, Ortholist fails to identify *cep-1* as a *TP53* ortholog due to the genes’ low sequence homology. However, *cep-1* has been proven as a bona fide functional ortholog of *TP53* (Derry et al., 2001). Despite the likely underestimate, my brief analysis shows that *C. elegans* carries orthologs of about three-quarters of human cancer driver genes, pointing to a high degree of conservation.

**Table 1.**
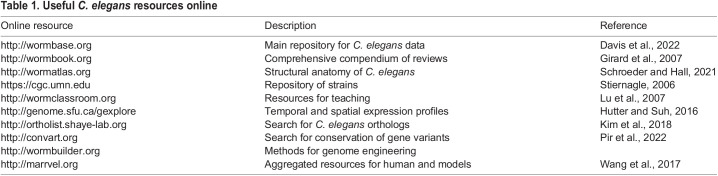
Useful *C. elegans* resources online

Therefore, the conservation of genes and biological mechanisms support the use of *C. elegans* in cancer research. In this Review, I use the hallmarks of cancer as a blueprint to update, refresh and reinforce the value of *C. elegans* as a model to investigate cancer.

## Cancer hallmarks in *C. elegans*

In 2000, Hanahan and Weinberg published ‘The hallmarks of cancer’ (Hanahan and Weinberg, 2000), an iconic Review that was embraced by cancer researchers worldwide. This article discusses “six essential alterations in cell physiology that collectively dictate malignant growth”: sustaining proliferative signaling, evading growth suppressors, resisting cell death, enabling replicative immortality, inducing angiogenesis, and activating invasion and metastasis. These were the first hallmarks of cancer, but the authors anticipated that “the search for the origin and the treatment of this disease will continue over the next quarter century by adding further layers of complexity”. Their prediction was correct, and the same authors published ‘Hallmarks of cancer: the next generation’ in 2011 (Hanahan and Weinberg, 2011), adding two emerging hallmarks, reprogramming of cellular energetics and avoiding immune destruction, and two enabling characteristics, genome instability and inflammation. In 2022, Hanahan published the latest iteration in the series, ‘Hallmarks of cancer: new dimensions’ (Hanahan, 2022), consolidating the previous core of eight hallmarks and two enabling characteristics, and proposing two new hallmarks, unlocking phenotypic plasticity and senescent cells, and two new emerging characteristics, epigenetic reprogramming and polymorphic microbiomes. In this Review, I use this explicit classification to comment on some of the numerous *C. elegans* studies that have contributed to a better understanding of ten hallmarks of cancer, underscoring the value of this model organism to disentangle the complexity of cancer mechanisms.

### Hallmark 1: sustaining proliferative signaling

Cell cycle progression drives cellular proliferation in a similar manner in all eukaryotes, and most of the cell cycle components are conserved from yeast to humans. Core elements of the cell cycle, which have been extensively studied in *C. elegans* (Kipreos and van den Heuvel, 2019), are not commonly mutated in cancer, probably because they are too essential to tolerate mutations hampering their functions and still permit cell survival. However, core cell cycle components are often deregulated by diverse mechanisms, such as altered proliferative signals. In *C. elegans*, hyperplasia phenotypes due to excessive cellular proliferation can be produced in different ways, such as by RNA interference (RNAi)-mediated inactivation of the cell cycle inhibitor gene *cki-1*/*CDKN1B* (Boxem and Van den Heuvel, 2002) or by gain-of-function mutations in the *CDC25A* oncogene ortholog *cdc-25.1* (Clucas et al., 2002).

**Fig. 2.**
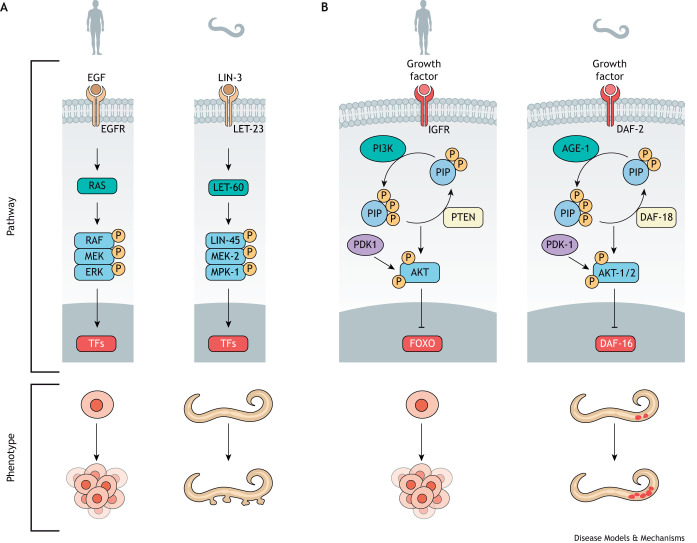
**Sustaining proliferative signaling pathways conserved from *C. elegans* to mammals.** Sustained proliferative signaling is one of the original hallmarks of cancer defined in Hanahan (2022), but these pathways have been the subject of intense research, and significant therapeutic success, for far longer. (A,B) A simplified schematic of two well-known proliferative signaling pathways that are often dysregulated in cancer, RTK-RAS/MAPK (A) and PI3K (B), paralleling the analogous mammalian and *C. elegans* pathways. Sustained signaling through these pathways increases cell proliferation in human cells and causes diverse phenotypes in *C. elegans*. LET-60/HRAS activation causes a protruding vulva phenotype, whereas sustained AGE-1/PI3K signaling prompts exit from quiescence in some postembryonic cells. P, phosphorylation; PIP, phospholipid; TFs, transcription factors.

Signaling pathways promoting cell cycle entry, like the receptor tyrosine kinase (RTK)-RAS/MAPK cascade, are well conserved and tightly regulated in metazoans. Elevated expression or gain-of-function mutations in the RTK-RAS/MAPK pathway, or loss-of-function mutations in its inhibitors, promote cancer. Researchers have characterized *C. elegans* orthologs of the RTK-RAS/MAPK core components (Fig. 2A), from the receptors and their ligands to the ETS transcription factors responsible for the transcriptional output of the pathway that drives proliferation. These core components participate in many developmental processes, interacting with other proteins and pathways in a cell-type-specific manner. Alteration of this pathway during worm development can result in overt phenotypes, such as a protruding vulva, or less obvious ones, such as modification of cell fates or defective cell migration (Sundaram, 2006). Interestingly, LET-60/HRAS expression is regulated by the conserved *let-7* microRNA (miRNA) family, which is also expressed in humans. Human RAS family genes contain multiple *let-7* complementary sites at their 3′ untranslated regions. Downregulation of *let-7* results in RAS overexpression and a deregulated progression through the cell cycle (Johnson et al., 2005; 2007).

Conveniently, the postembryonic development of the vulva, an epithelial aperture on the nematode's ventral side that allows mating and egg laying, is an amenable process for studying the function of RAS. Loss of LET-60/HRAS function causes a vulvaless (Vul) phenotype, whereas gain-of-function mutations produce multivulva (Muv) animals. Although these phenotypes are provoked by alterations of cell fates and do not represent overproliferation, the Vul and Muv models have been widely used to identify other components of the RTK-RAS/MAPK pathway (Sundaram, 2013), cross-talks with other signaling cascades (Lee and Yoon, 2017; Corchado-Sonera et al., 2022) and chemical inhibitors (Reiner et al., 2008; Schmid et al., 2015; Ji et al., 2019; van der Hoeven et al., 2020). Thus, genetic screens using the Muv phenotype have allowed the identification of RTK-RAS/MAPK components in *C. elegans* first, like KSR-1, the ortholog of human KSR1 and KSR2 (Kornfeld et al., 1995; Sundaram and Han, 1995), and SEM-5, the ortholog of human GRB2 (Clark et al., 1992). Besides genes and molecules, vulva development as a phenotypic readout has also contributed to the identification of other factors influencing LET-60/HRAS activity, such as hypoxia (Maxeiner et al., 2019), oxidative stress (Kramer-Drauberg et al., 2020), starvation (Grimbert et al., 2018) or alternative polyadenylation (Subramanian et al., 2021).

The phosphatidylinositol 3-kinase (PI3K; PIK3) pathway also promotes proliferative signals activated by extracellular stimuli such as insulin or growth factors (Fig. 2B). This pathway is heavily deregulated in cancer, either via aberrant activation of oncogenes, like PI3K itself and its downstream kinase AKT, or via the loss of tumor suppressors such as the lipid phosphatases INPP4B and PTEN. The axis of this pathway in *C. elegans* is commonly studied in the context of aging and metabolic signaling (Fig. 2B). Interestingly, the PTEN ortholog DAF-18 is required to maintain the quiescence of some postembryonic cells. Germ cell precursors and the mesoblast (M) cell begin to proliferate after hatching in the presence of food. If food is not present, these cells remain quiescent in wild-type *C. elegans*, but divide in DAF-18/PTEN mutants (Fry et al., 2021; Chen et al., 2022). This observation demonstrates that worms with hampered DAF-18/PTEN activity or a hyperactivated PI3K are an excellent model to study the regulatory signaling that modulates the quiescent-to-proliferative state transition. Conveniently, a humanized *C. elegans* model (Box 2) of PTEN mutations has been developed by substituting the endogenous *daf-18* gene with human *PTEN*, which rescues multiple *daf-18* mutant phenotypes (McDiarmid et al., 2018).

**Box 2. CRISPR editing and humanization**

For CRISPR-based gene editing in *C. elegans*, reagents are injected into the germline of young adult hermaphrodites, which consists of two symmetric U-shaped tubular structures with hundreds of germ nuclei that form a syncytium. These nuclei later form oocytes that are fertilized when they cross the spermatheca. Thus, by injecting the cocktail of CRISPR reagents in a germline arm, hundreds of nuclei are potentially exposed to gene editing. A single microinjection can produce dozens of edits, but normally 15-20 animals are microinjected to secure the experiment, particularly if the intent is not to provoke mutation via error-prone repair but to induce a precise genome edit. Importantly, the short life cycle (embryo to adult in 3-5 days) and the self-fertilization of hermaphrodites allows for obtaining homozygous edits in 2 weeks.

CRISPR reagents can be injected as robust ribonucleoproteins composed of Cas nucleases and synthetized guide RNAs (gRNAs), or as plasmids that are then expressed in the targeted cells (Nance and Frøkjær-Jensen, 2019). Moreover, researchers have engineered *C. elegans* strains that express Cas9 (Schwartz et al., 2021), including a strain that expresses a minimal-protospacer adjacent motif (PAM) Cas9 variant in the germline (Vicencio et al., 2022). Using such strains reduces the cost of gene-editing experiments.

In the context of cancer research in worms, CRISPR can be used to mutate genes of interest, generating precise deletions or missense mutations in conserved amino acids, to investigate their biological functions. Whereas a complete deletion results in a null allele, partial loss-of-function alleles can be obtained by microdeletions or by tagging the gene with a fluorescent reporter that can, in certain cases, hamper its function. CRISPR can also be used for producing excess-of-function alleles by inserting additional copies of any gene into precise genomic sites to model copy-number gains often seen in human cancer genomes (Yoshina et al., 2015; Mouridi et al., 2022; Malaiwong et al., 2023).

Endogenous fluorescent reporters are valuable genetic tools (Paix et al., 2015; Vicencio et al., 2019) for studying the activity of a specific pathway or oncogenic process. Reporter worm strains can be used in genetic screens, like classic mutagenesis-, RNA interference (RNAi)- or CRISPR-knockout-based approaches (Yang et al., 2020).

Finally, cancer-related genes in *C. elegans* can be partially or fully humanized by replacing the worm gene with its human ortholog using CRISPR, as demonstrated with *daf-18*/*PTEN* (McDiarmid et al., 2018). Once the edit has been confirmed, researchers need to assess whether the human gene, or part of it, is functional in the worm. If the loss of the endogenous worm gene causes a phenotype that is rescued by the human(ized) replacement (in the same locus or a different one), this means that the human ortholog is functional, and therefore effects of cancer-related mutations can be assessed in living worms.

In the context of the PI3K pathway, the nutrient-sensing serine/threonine kinase mTOR regulates developmental progression, and promotes tumor growth and metastasis in diverse cancer types. The *C. elegans* protein LET-363 is an ortholog of mTOR, and DAF-15 and RICT-1 correspond to the two mTOR interactors in mammalian cells, RAPTOR (RPTOR) and RICTOR, respectively. Confirming the conserved role of mTOR as a developmental regulator, *let-363* inactivation by RNAi or mutations affects developmental processes, including germ cell proliferation, exit from quiescence or embryonic development (Keith Blackwell et al., 2019). Unfortunately, the most studied and clinically used mTOR inhibitor rapamycin, which forms a complex with the chaperone FKBP12 to inhibit the FRB domain of mTOR (Huang et al., 2003), has a limited impact on *C. elegans* growth and development, although it increases its lifespan (Robida-Stubbs et al., 2012). Despite the limitations of rapamycin in worms, studies on LET-363/mTOR signaling in *C. elegans* keep providing layers of information on its functions. As an example, the mitochondrial nuclease ENDOG suppresses the mTOR pathway to promote autophagy (Wang et al., 2021), a conserved pathway for which impairment is also related to cancer (see ‘Hallmark 3: resisting cell death’ section).

Finally, I must mention in this section the germline tumors produced by elevated activation of GLP-1/Notch in the *C. elegans* distal tip cells, which are somatic cells (Berry et al., 1997). This overt proliferative phenotype has been used to identify new genetic interactions with the Notch pathway (Singh et al., 2021).

Aside from investigating individual proliferative signaling pathways, genetic analyses in *C. elegans* can advance the study of pathway cross-talks, as exemplified by the finding that the PI3K signaling repressor DAF-18/PTEN also acts as a negative regulator of RTK-RAS/MAPK signaling during vulva development (Nakdimon et al., 2012). The well-characterized genetics of *C. elegans* allow researchers to understand the complexities of cancer-related pathway crosstalk in a tractable and relatively simple system.

### Hallmark 2: evading growth suppressors

Tumor suppressor genes are like brakes against the onset and progression of cancer. The two prototypical tumor suppressors, the chromatin-remodeling protein retinoblastoma (Rb; RB1) and the transcription factor P53 (TP53), correspond to *C. elegans* LIN-35 and CEP-1, respectively. LIN-35/Rb is a hub regulator of diverse pathways upon extracellular and intracellular signals. Complete deletion of the *lin-35* ortholog in the soil nematode *Caenorhabditis briggsae* produces very sick animals (Burton et al., 2021), suggesting that a full *lin-35* deletion might not be viable in *C. elegans*. However, *C. elegans* with an early stop codon (allele *n745*) in *lin-35* are viable, and this mutation can be considered a putative null allele, at least for some of the LIN-35 functions (Lu and Horvitz, 1998). *lin-35(n745)* animals present diverse alterations, such as upregulation of genes otherwise repressed by the DREAM chromatin remodeling complex (Goetsch et al., 2017), sensitivity to RNAi (Wang et al., 2005) or additional intestinal cells (Boxem and Van den Heuvel, 2001). Functional redundancy is a remarkable feature of LIN-35/Rb, highlighting its capacity to influence different genetic pathways via synthetic genetic interactions with other mutations. The synthetic Muv (synMuv) phenotype is a clear example. Although mutations in single synMuv genes, one of which being *lin-35* itself, do not cause a Muv phenotype, specific combinations of at least two mutations do (Ceol et al., 2006). Such capacity for genetic interactions and clear phenotypic readouts have facilitated forward and reverse genetic screens that used traditional mutagenesis and RNAi, respectively, to identify functional interactions of LIN-35/Rb with multiple cellular and developmental processes (Fay et al., 2002; Thomas et al., 2003; Ceron et al., 2007).

P53 is mutated in about half of human cancers (Perri et al., 2016). Its role is to sense different stresses, including DNA damage, and trigger a transcriptional response to repair the damage or to induce apoptosis. In humans, *TP63* and *TP73* are the other two members of the gene family. They seem to be less commonly implicated in cancer but have overlapping functions in distinct tissues that complicate functional studies of *TP53*. Invertebrates present a single member of the p53 family containing an evolutionary conserved p63-like domain structure (Rutkowski et al., 2010). *cep-1* is the sole member of the family in *C. elegans*. Owing to its low sequence but high structural homology, CEP-1's orthology to p53 in regulating DNA-damage-induced apoptosis and genome stability was identified a bit late, in 2001 (Derry et al., 2001; Schumacher et al., 2001). *cep-1* loss-of-function alleles do not cause obvious developmental phenotypes, just mild defects that do not compromise viability. Interestingly, *cep-1* mutants do not accumulate mutations in the genome under unchallenged growth conditions. However, introducing a *cep-1* mutation into strains with severe DNA repair defects increases the rate of mutagenesis (Meier et al., 2021). Upon ultraviolet (UV) irradiation, CEP-1 is required for DNA damage-induced apoptosis and cell cycle arrest in the germline (Derry et al., 2007) but not in somatic cells, which do not express DNA damage sensors such as ATM-1*/*ATM (Vermezovic et al., 2012). Still, some studies suggest a role for CEP-1 in somatic cells, contributing to halt the cell cycle in embryonic cells upon impairments of the DNA damage response signaling (Moser et al., 2009). Beyond Rb and P53, *C. elegans* can be used for investigating the functions of other tumor suppressors, such as the CYLD deubiquitinase, which stabilizes P53 activity in the DNA damage response (Fernández-Majada et al., 2016).

### Hallmark 3: resisting cell death

Apoptosis is a highly regulated type of programmed cell death that is typically inhibited in cancer. Moreover, certain tumors can become resistant to apoptosis induced by chemotherapy. Therefore, anti-apoptotic proteins are potential targets for therapies. Interestingly, the core components of apoptotic pathways are not only evolutionarily conserved but were first identified in *C. elegans* (Horvitz, 2003). This discovery was expedited for two features of worms: (1) the transparency of the animal, which allows the observation of individual cells and apoptotic corpses, facilitated the description of invariant somatic lineages and the stereotyped pattern of apoptotic events (Sulston and Horvitz, 1977; Sulston et al., 1983); (2) programmed cell death is not essential for the viability of the organism (Ellis and Horvitz, 1986), allowing efficient identification of genes required for normal patterns of apoptosis during development.

In the canonical *C. elegans* apoptosis pathway, transcriptional activation of *egl-1*, which encodes a BH3-only protein, inhibits the antiapoptotic CED-9/BCL2. This permits the activation of the proapoptotic CED-4/APAF1. CED-4 activity is required for CED-3/Caspase to finally execute the apoptotic cell death. Importantly, not only is the core apoptotic pathway conserved, but *C. elegans* also carries orthologs to mammalian genes implicated in its regulation (Conradt et al., 2016; Wei et al., 2020) and in the recognition and clearance of apoptotic cell corpses (Lukácsi et al., 2021) (Table 2).

**Table 2.**
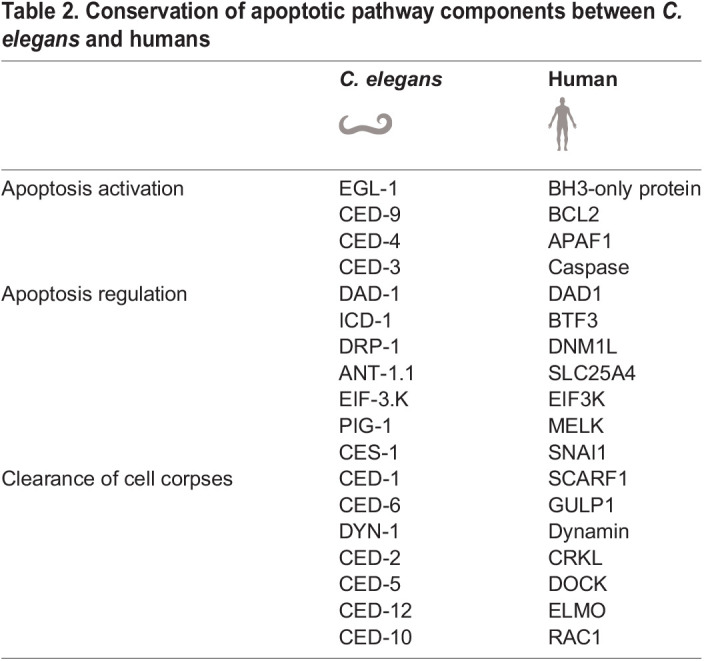
Conservation of apoptotic pathway components between *C. elegans* and humans

Exposure of *C. elegans* to DNA damage via UV, γ-irradiation or the chemotherapeutic drug cisplatin can induce ectopic apoptotic cell corpses (Gartner et al., 2000; García-Rodríguez et al., 2018). Thus, *C. elegans* is a suitable model for studying DNA damaging agents and apoptosis execution in an *in vivo* setting. In addition, worms have helped researchers describe other factors producing apoptotic corpses, such as the inactivation of splicing components (Rubio-Peña et al., 2015).

Moreover, cell death inhibitors have been identified in *C. elegans* screens. Although they may not have therapeutic interest, these findings contribute to a better understanding of factors and pathways involved in cancer cells' resistance to cell death (Schwendeman and Shaham, 2016; Brantley et al., 2017).

Also related to resistance to cell death and cancer, the regulated process of autophagy promotes cell survival through metabolic rearrangements that imply lysosome-dependent degradation of organelles. Genes involved in autophagy and their functions are evolutionary conserved from worms to mammals (Wong et al., 2020). The *C. elegans* research community has well-established tools to study autophagosomes, the key spherical structures in autophagy with double-layer membranes that degrade cellular components (Peña-Ramos and Zhou, 2022). Moreover, the functional interconnection between autophagy and apoptosis pathways can be investigated in *C. elegans* (Ploumi et al., 2023).

### Hallmark 4: enabling replicative immortality

Telomerase maintains telomeres at chromosome ends, which is important for sustained replications, but which also limits the replicative capacity of normal cells. However, cancer cells have developed mechanisms to preserve telomeric DNA to avoid cell cycle exit and senescence, effectively rendering them immortal. Although mammalian telomeres consist of TTAGGG repeats, the *C. elegans* telomere sequence is TTAGGC and repeats span for 4-9 kb at the ends of chromosomes (Wicky et al., 1996). *C. elegans* telomeres can also form G-quadruplex structures (Marquevielle et al., 2022), supporting the similarities between telomeres of mammals and worms. Lack of *trt-1*, the *C. elegans* gene encoding telomerase, causes sterility after 14-18 generations (Meier et al., 2006). Thus, telomerase activity is necessary to maintain immortality in *C. elegans* germ cells. Interestingly, telomerase-independent mechanisms for protecting chromosomal ends were identified among *trt-1* mutants that remained fertile for more than 18 generations (Seo et al., 2015). Thus, similar to cancer cells, *C. elegans* germ cells counteract the shortening of telomeres.

The state of *C. elegans* telomeres can be determined by fluorescence *in situ* hybridization to investigate genes or other factors influencing telomere biology (Seo and Lee, 2016). Several orthologs of human protection of telomeres (POT) proteins have been identified in *C. elegans*, including POT-1, POT-2, POT-3 and MRT-1 (Shtessel et al., 2013). Telomere length variations among natural isolates of *C. elegans* correlate with *pot-2* variants, supporting the existence of genetic variants that favor inter-individual diversity of telomere lengths (Cook et al., 2016). Interestingly, these differences do not correlate with changes in progeny number or longevity. Moreover, the short life cycle of *C. elegans* has facilitated a study showing that altered levels of POT-1 and POT-2 foci, which are POT protein aggregates at telomeres, in certain genetic backgrounds display transgenerational epigenetic inheritance (Lister-Shimauchi et al., 2021).

Another protein involved in telomere metabolism is the WRN helicase, which is also involved in replication arrest recovery and DNA repair (Crabbe et al., 2004). Mutations in the *WRN* gene cause Werner syndrome, a rare premature aging syndrome associated with genome instability and an increased incidence of cancer. WRN-1, the *C. elegans* ortholog for WRN, is part of DNA damage response signaling pathways and is involved in the repair of double-stranded DNA breaks caused by cytotoxic agents, including cisplatin (Hyun et al., 2016; Ryu and Koo, 2017) (Box 3). Thus, *C. elegans* can help to study alternative functions of genes related to maintaining the integrity of telomeres and DNA as a whole, and the connection between these processes and unlimited replicative potential.

**Box 3. Investigating cancer therapies in *C. elegans***

Cancer is not a single disease. The term spans multiple diseases arising in distinct organs and cells, altering diverse cellular pathways and genes. However, this is not the only feature complicating cancer treatments. Cancer cells commonly develop mechanisms of resistance against therapies, forcing the use of alternative treatments. Therefore, oncologists need a wide collection of therapeutic approaches. The implementation of new cancer therapies in the clinic and the optimization or repurposing of existing ones are long journeys that *C. elegans* can help shorten.

**1. First-line chemotherapies**

Chemotherapeutic agents used as first-line cancer treatments present low specificity and therefore efficiently control cell proliferation in most organisms. When using these agents, there are two main concerns: toxicity to normal cells and the onset of cellular resistance in cancer cells. Studies in *C. elegans* can help reduce these concerns by helping understand the underlying mechanisms.

Cisplatin is probably the most common chemotherapeutic in the treatment of solid tumors. Depending on the dose, cisplatin causes diverse phenotypes in *C. elegans*, including reduced mobility, sterility or body size (Hemmingsson et al., 2010; García-Rodríguez et al., 2018). These phenotypes can be used to identify genes or molecules that affect the therapeutic impact of cisplatin (Piulats et al., 2018; Martínez-Fernández et al., 2022). Similarly, genes affecting sensitivity to 5-fluorouracil have been identified in worms (Kim et al., 2008). *C. elegans* has also been used to study the variability in response to bleomycin (Brady et al., 2019), the genomic lesions caused by mitomycin (Tam et al., 2015), and the effect on the microbiota and toxicity caused by floxuridine (Ke et al., 2020).

**2. Targeted therapies, humanization of drug targets and synthetic lethality**

More specific cancer drugs that affect certain oncogenic pathways or a single protein can also be investigated in nematodes. In principle, if a small molecule has an impact on a signaling pathway such as Wnt, Notch or RAS in worms, it will probably have an impact on the homologous human pathway (Kobet et al., 2014) and could be chemically modified for a more efficient action in human cells. For example, known RAS inhibitors have been proven effective in inhibiting the RAS-dependent multivulva phenotype in *C. elegans* (Reiner et al., 2008; Bae et al., 2012; van der Hoeven et al., 2020), and small molecules modulating the RAS pathway were identified in worms (Schmid et al., 2015).

If an inhibitor does not work in *C. elegans* because the molecular dock of the targeted protein is not conserved, such region can be humanized, producing a strain sensitive to the drug of interest. As an example, a specific region of *sftb-1/SF3B1* was humanized by CRISPR to render a worm strain sensitive to splicing inhibitors, which are of therapeutic interest in cancer (Serrat et al., 2019). This strain can then serve as a platform for studying chemical derivatives of such drugs that have higher efficacy and less toxicity in animals, and eventually in the clinic.

Finally, the classic strategy of identifying synthetic lethal interactions to find novel therapeutic targets, particularly for cancers that are driven by loss of tumor suppressors rather than by activation of a directly targetable oncogene, has also been explored in *C. elegans* (Fay et al., 2002; Ceron et al., 2007; McLellan et al., 2012; Serrat et al., 2019; Bellelli et al., 2020).

**3. Drug discovery**

*C. elegans* cultures can be expanded to produce large populations of worms allowing high-throughput screens of small-molecule libraries (Giunti et al., 2021). To achieve reliable read-outs from such drug discovery screens, researchers need a homogenous population of worms, which can be achieved by the simple method of synchronizing animals with bleach and sodium hydroxide (Porta-de-la-Riva et al., 2012). Once the worms are synchronized, small molecules can reach their cells through the intestine if mixed with the food or by crossing the cuticle (Zheng et al., 2013). Thus, several *C. elegans* strains carrying mutations affecting cuticle integrity have been reported to have increased drug absorption. Using these strains in drug screens reduces potential false-negative hits due to insufficient doses reaching the target cells. In laboratory conditions, *C. elegans* is commonly fed with genetically well-characterized strains of *Escherichia coli*. To avoid concerns about the capacity of bacteria to metabolize the screened drugs, *C. elegans* can be fed with dead bacteria or axenic liquid media.

Whereas drug screens in other *C. elegans* disease models commonly look for molecules that alleviate the disease-related phenotype (Moy et al., 2006; Kukhtar et al., 2020), cancer-related screens may need to search for synthetic lethality events. In either case, the main factor in rendering a screen valuable is to have a clear phenotypic readout.

### Hallmark 5: activating invasion and metastasis

In the early 2000s, Sherwood and colleagues described a *C. elegans* developmental process that models cell invasion through basement membranes, which is a key step in metastasis (Sherwood and Sternberg, 2003). The authors used a labeling system to identify and track the vulva and the anchor cell, a specialized cell of the somatic gonad. In the Larva 3 stage, the anchor cell invades the vulva epithelium after disruption of the basement membrane, connecting the uterus to the developing vulva (Sherwood and Sternberg, 2003). A follow-up study by the same group identified *fos-1*, similar to the human transcription factor FOS, as essential for the disruption of the basement membrane. FOS-1 promotes invasion by regulating the expression of several genes, including some related to the extracellular matrix that are conserved in humans (Sherwood et al., 2005).

Subsequently, an RNAi screen of transcription factors essential for anchor cell invasion of the basement membrane identified NHR-67. This transcription factor upregulates *cki-1*/*CDKN1B* levels, arresting the cell cycle before the invasion (Matus et al., 2015). This shows that the proliferative and invasion states are incompatible in the anchor cell, which opened an interesting view of tumor cells, in which the cellular programs to proliferate or invade may be mutually exclusive to some extent. The aforementioned anchor cell invasion model has recently helped identify other elements of the network regulating invasion, such as EGL-43 (Deng et al., 2020) or components of the chromatin-remodeling SWI/SNF complex (Medwig-Kinney et al., 2020; Smith et al., 2022).

Another angle from which to investigate invasion and metastasis in nematodes is through the Metastasis-associated protein (MTA) family. In humans, the MTA1, MTA2 and MTA3 proteins are subunits of diverse chromatin-remodeling complexes, and their expression in tumors correlates with a poor prognosis (Kumar and Wang, 2016). *C. elegans* carries two MTA proteins, LIN-40 and EGL-27. LIN-40 is part of the nucleosome remodeling and deacetylase (NuRD) complex involved in nucleosome remodeling and chromatin deacetylation to regulate cell fates (Chen and Han, 2001), whereas EGL-27 influences gene expression in morphogenesis, stress response and the DNA damage response (Xu and Kim, 2012; Mueller et al., 2014).

Because metastasis requires cell migration, this cellular process is another bridge to connect *C. elegans* research with metastasis (Stuelten et al., 2018). In this context, the conserved family of ADAMTS metalloproteases, responsible for regulating extracellular matrix and potential therapeutic targets, has been studied in *C. elegans* (Ismat et al., 2013).

### Hallmark 6: deregulating cellular metabolism

Tagged as an ‘emerging hallmark’ in 2011, reprogramming of cellular energetics has been designated as a core cancer hallmark in 2022 (Hanahan, 2022). In the presence of oxygen, normal cells use glycolysis to produce ATP, NADH and pyruvate in the cytosol, which is later oxidized to carbon dioxide in the mitochondria, generating additional ATP in the process. Cancer cells present an increase in glucose uptake, but they favor the production of lactate from pyruvate, even in the presence of oxygen, which is a less efficient way of producing ATP. This metabolic reprogramming, the Warburg effect, has been known for more than a century (reviewed in DeBerardinis and Chandel, 2020), and is even stronger in the hypoxic conditions found in many tumors. Whether there is any benefit to reprogramming toward a much less efficient production of ATP is not well understood yet. Because some embryonic tissues have a Warburg-like metabolism, it is hypothesized that such energetic reprogramming allows the use of glycolytic intermediates in biosynthetic pathways required for cell proliferation (Krisher and Prather, 2012). Conveniently for metabolic studies, *C. elegans* ATP levels and mitochondria respiration can be measured in individuals or small populations (Koopman et al., 2016). Similarly to human cell lines, treating worms with arsenic causes a Warburg effect, which has been used to identify components of glycolysis and electron transport chain complexes (Zhao et al., 2013; Luz et al., 2016). Besides this arsenic-induced model, a Warburg-like aberrant glycolysis has been studied in a *C. elegans* strain carrying mutant *sdhb-1* that mimics a missense mutation of the human succinate dehydrogenase associated with rare heritable neuroendocrine cancers (Saskői et al., 2020). Another glycolytic shift reported in *C. elegans* is the increase of lactate after activating small-conductance calcium-activated K^+^ (SK) channels, which confers resistance to oxidative stress and neuroprotection (Krabbendam et al., 2020).

Thus, although stable tumor-like structures are not well established in *C. elegans*, factors influencing the metabolic switch from mitochondrial respiration to lactate-producing glycolysis, as well as other aspects of cancer-specific metabolic rewiring, can be investigated in this model.

### Hallmark 7: genome instability and mutation

Some phenotypes related to genome instability are easily observable in *C. elegans*. As an example, a high prevalence of males (X/0) in the progeny of hermaphrodites (X/X) indicates defects in meiotic chromosome segregation and the onset of aneuploidy. The worm's transparency facilitates the observation of stained chromosomes, allowing the detection of mitotic artifacts (Porta-de-la-Riva et al., 2012), which are also a common occurrence in cancer cells.

A common methodology to identify genes involved in genome instability is to screen for sensitivity to DNA damage. This approach has identified components for distinct repair mechanisms in *C. elegans*. Most of the genes related to DNA repair mechanisms are conserved, including *atl-1*/*ATR*, *atm-1*/*ATM*, *brc-1*/*BRCA1/2*, *chk-1*/*CHEK1*, *chk-2*/*CHEK2*, *cku-70*/*XRCC6*, *dog-1*/*BRIP1*, *msh-2*/*MSH2* and *rad-51*/*RAD51*. These genes are included in a recent compilation and review in WormBook (Gartner and Engebrecht, 2022).

The mechanisms that keep the frequency of spontaneous mutations low are crucial for the correct development of an organism. However, cancer cells often present elevated mutational rates that may favor their clonal expansion. Cancer cells accumulate mutations by disrupting DNA repair, bypassing cell cycle checkpoints or inactivating the cellular surveillance systems that trigger apoptosis or senescence upon excessive DNA damage. In a recent study (Meier et al., 2021), more than 20 *C. elegans* DNA repair mutants were maintained for several generations, and their genomes were sequenced to observe that these animals displayed a more than twofold increase in the number of diverse mutation types, including deletions, structural variants and base substitutions. Interestingly, *brc-1*/*BRCA1* and *rad-51*/*RAD51* mutants showed an increase in the number of mutations of all types (Meier et al., 2021).

Besides the implication of DNA replication or DNA damage response in genome instability, proteins involved in RNA processing and export, such as components of the THSC/TREX-2 complex, have also been linked to transcription-associated genome instability in human cells and in worms (Bhatia et al., 2014; Zheleva et al., 2021).

Finally, as an example of how *C. elegans* can open new lines of thought in cancer research, functional interactions between DNA repair proteins and chromatin remodelers such as histone methyltransferases (Padeken et al., 2019; Yang et al., 2019) or components of the DREAM complex (Bujarrabal-Dueso et al., 2023) have been observed. These findings further expand the complexity of factors influencing the mutational rate, a key process in cancer.

### Hallmark 8: nonmutational epigenetic reprogramming

Cancer cells can use other strategies besides mutations or chromosomal alterations to reprogram gene expression. To adapt to a changing microenvironment caused by hypoxia or other cellular stresses, cells can modify their epigenome to activate a favorable gene expression program, mainly via DNA methylation, histone modifications or expression of small RNAs. DNA methylation on cytosines (5mC), mostly in CpG dinucleotides, is a common repressive mark in mammals (Greenberg and Bourc'his, 2019). However, *C. elegans* does not present 5mC and lacks orthologs to mammalian cytosine DNA methyltransferases. The presence of other DNA methylation marks in *C. elegans*, such as 6mA, has been controversial and does not represent a major drive for reprogramming gene expression in worms (O'Brown et al., 2019). However, histone modifications, and the enzymes responsible for them, are well conserved. When encountering an altered environment, like the presence of pathogens, osmotic stress, exposure to chemicals or a lipid-rich diet, worms reprogram their epigenome to acquire new gene expression programs, and these programs maintain a transgenerational inheritance (Camacho et al., 2018; Özdemir and Steiner, 2022; Wan et al., 2022). Small RNAs also participate in modifying the epigenetic landscape in *C. elegans* and are regulated by environmental changes (Houri-Zeevi et al., 2021). Indeed, our understanding of small RNAs as regulatory molecules of the genome initiated with the work of *C. elegans* researchers Andrew Fire and Craig Mello, who received a Nobel prize for their discovery of RNAi (Fire et al., 1998). Finally, next-generation sequencing technologies allow the study of RNA modifications, such as methylated and thiolated ribonucleotides, which have been identified in RNAs from human cancers and nematodes (Barbieri and Kouzarides, 2020; Li et al., 2020).

### Hallmark 9: polymorphic microbiomes

In the past few years, many articles have described the association between microbial communities and response to cancer therapies. In particular, the impact of the digestive tract microbiota on colorectal cancer is well established (Sears and Garrett, 2014). The microbiota associated with the digestive tract of animals can be studied in the *C. elegans* gut (Douglas, 2019). Thus, *C. elegans* modeling can address diverse questions related to microbiota, such as how microbes affect the host transcriptome (Gómez-Orte et al., 2017), the impact of microbial byproducts on the host organism (Venzon et al., 2022) or the influence of host genetic backgrounds in microbiome selection (Zhang et al., 2021).

To study the natural components of the *C. elegans* microbiome, researchers have established CeMbio, a resource for the community that includes a collection of 12 bacteria from nine different families (Dirksen et al., 2020). In addition, *C. elegans* can be infected with human enteric bacterial pathogens to study host–pathogen interactions (Walker et al., 2021). As an example of studies related to the microbiome–cancer axis, certain strains of *Enterococcus faecalis* are potentially oncogenic because of their capacity to produce reactive oxygen species (ROS) and induce DNA damage, and *C. elegans* have been used to study *Enterococcus* infections (Yuen and Ausubel, 2014; Sim and Hibberd, 2016). A separate study demonstrated that *Rhizobium* also produces ROS that induce DNA damage and mitotic defects in the *C. elegans* intestine (Kniazeva and Ruvkun, 2019).

Another aspect of the impact of microbiota in cancer that can be studied in the worm is how certain microbes' metabolism can influence responses to chemotherapy (García-González et al., 2017). The presence of distinct bacterial strains in *C. elegans* correlates with different toxicities caused by the chemotherapeutic agent 5-fluorouracil (Nguyen et al., 2022), and *C. elegans* have been used to study how human microbiome bacteria reduce the toxic effect of the anticancer drug doxorubicin (Yan et al., 2018). Thus, although the immune-regulatory effect of the mammalian microbiome (Gharaibeh and Jobin, 2019) would be more difficult to investigate in *C. elegans*, many questions related to microbiome interactions with the host and chemotherapeutic agents can be addressed in worms.

### Hallmark 10: unlocking phenotypic plasticity

Most terminally differentiated cells stay in a nonproliferative state. Thus, a potential cancer cell needs to avoid differentiation or acquire its replicative capacity through dedifferentiation or transdifferentiation to a different cell type compatible with tumorigenesis. The stereotyped cell lineage and precise cellular map of *C. elegans* can contribute to the investigation of genes and pathways related to processes to maintain or acquire a cellular state compatible with proliferation.

In *C. elegans*, muscle precursors cells can skip differentiation and keep proliferating when the activity of cell cycle entry inhibitors and of SWI/SNF components is hampered by mutations, RNAi or lineage-specific gene inactivation (Ruijtenberg and Van Den Heuvel, 2015). A follow-up study by the same group demonstrated that partial inactivation of SWI/SNF complex components can induce overproliferation, but a minimal activity of these SWI/SNF proteins is required to maintain essential cellular functions (van der Vaart et al., 2020). In other words, depending on the level of inactivation, reduced SWI/SNF activity can either stimulate or inhibit proliferation. These studies set *C. elegans* as an excellent model to further investigate SWI/SNF in diverse cancer types and to explore vulnerabilities of tumors harboring mutations in SWI/SNF subunits (Mittal and Roberts, 2020; Wanior et al., 2021).

Diverse research tools are available to study the plasticity of *C. elegans* cells. Advances in single-cell omics allow the identification of differentiation markers in specific *C. elegans* cells or lineages, and therefore the study of transcriptomic profiles associated with proliferative capacities (Cao et al., 2017). Besides neurons, vulval cells have been extensively used in studies of genes expressed in specific postmitotic cells (Gupta et al., 2012). Moreover, cell cycle sensors allow *C. elegans* researchers to track entry into the cell cycle and therefore the transition from a nonproliferative to a proliferative state *in vivo* (Van Rijnberk et al., 2017; Adikes et al., 2020).

Although *C. elegans* differentiated cells are highly resistant to modifying their fate (Coraggio et al., 2018), Polycomb mutants affecting histone methylation have the capacity to transdifferentiate, for example, from germ cells to neurons (Tursun et al., 2011), or dedifferentiate towards stemness. A study of the *C. elegans* nervous system has demonstrated that the expression of transcription factors determining neuronal identity restricts cellular plasticity through chromatin modifications, but their removal restores such plasticity (Patel and Hobert, 2017). Mutations in components of the chromatin-remodeling Rb pathway cause the ectopic expression of germ cell markers in somatic cells and the acquisition of germ cell features, a process that likely contributes to malignant transformation in Rb-mutant mammalian cells (Wang et al., 2005). Levels of Polycomb-regulated epigenetic marks regulating cell fate and proliferative signals like Notch signaling-inducing proliferation contribute to the delicate balance determining cell cycle re-entry in somatic cells (Coraggio et al., 2018). Interestingly, mutations in the components of polycomb repressive complex 2 were found in 25% of T-cell acute lymphoblastic leukemia, which is mainly driven by oncogenic activation of Notch signaling (Bardelli et al., 2021). Finally, by modifying in *C. elegans* the distribution of the chromatin mark H3K27me3, an alteration observed in certain pediatric gliomas, JNK signaling upregulation was identified as the cause of the ectopic replicative fate in germ cells that carry aberrant H3K27me3 marks (Delaney et al., 2019). Because JNK inhibition hampered proliferation in cell lines derived from such gliomas, this work produced a model for drug screens. Thus, despite the stereotyped and rigid lineages, the studies discussed here support the use of *C. elegans* as a model to investigate processes promoting plasticity between quiescent and proliferative cellular states.

## Conclusions

*C. elegans* is a valuable genetic model organism in which a malignant tumor has not been provoked yet. This causes some scepticism among researchers that use other systems to study cancer. However, extensive literature and a Nobel prize for discovering the core apoptotic pathway have validated the usefulness of *C. elegans* in cancer research. Showing that ten of the 14 iconic cancer hallmarks can be investigated in *C. elegans* may help to reconsider this powerful genetic model, free of ethical concerns, for solving key questions on oncogenic processes. This Review intends to provide a broad view of cancer mechanisms that can be explored in *C. elegans*, but it is not exhaustive, and I apologize to the many colleagues whose studies should, but could not, be cited due to space constraints. Other helpful reviews about cancer research in *C. elegans* were published in the past (Saito and Van den Heuvel, 2002; Kirienko et al., 2010; Kyriakakis et al., 2015). I decided to focus on ten hallmarks to underscore that *C. elegans* is not a magic tool to investigate all topics related to cancer, but it certainly is a precise tool to address specific questions on cancer mechanisms and uncover novel genetic interactions. Although *C. elegans* does not have a vascular system, it could potentially be used to investigate the 11th hallmark: inducing or accessing vasculature. In *C. elegans*, *pvf-1* encodes an ortholog of human/mammalian VEGF genes, and Ver genes (*ver-1*, *ver-3*, *ver-4*) share homology with mammalian genes encoding members of the VEGFR family, although they, of course, participate in different biological processes in worms (Dalpe et al., 2013; Luth et al., 2021). Far from being a closed concept, the hallmarks of cancers have been recently reviewed in the context of their interaction with the nervous system (Hanahan and Monje, 2023). In the search for models to address questions related to cancer–nerve interactions (Le and Oudin, 2023), *C. elegans* may prove to be an excellent model to investigate neuronal functions.

As well as providing valuable information on the biology of cancer, *C. elegans* can also help researchers understand the targets, mechanisms of action, and side effects of new and existing drugs (Box 3). As the statistician George Box once said, all models are wrong, but some are useful. Although not originally coined in the context of human disease models, this quote and its many variations have been widely adopted by our community. Like every other disease model, *C. elegans* is not perfect, but it can efficiently answer relevant questions that will advance our understanding of cancer and improve translation to the clinic to help patients.

## Supplementary Material

10.1242/dmm.050079_sup1 Supplementary information

## References

[DMM050079C1] Adikes, R. C., Kohrman, A. Q., Martinez, M. A. Q., Palmisano, N. J., Smith, J. J., Medwig-Kinney, T. N., Min, M., Sallee, M. D., Ahmed, O. B., Kim, N. et al. (2020). Visualizing the metazoan proliferation-quiescence decision in vivo. *eLife* 9, 1-74. 10.7554/ELIFE.63265 PMC7880687 33350383

[DMM050079C2] Bae, Y. K., Sung, J. Y., Kim, Y.-N., Kim, S., Hong, K. M., Kim, H. T., Choi, M. S., Kwon, J. Y. and Shim, J. (2012). An in vivo C. elegans model system for screening EGFR-inhibiting anti-cancer drugs. *PLoS ONE* 7, e42441. 10.1371/JOURNAL.PONE.0042441 22957020 PMC3434183

[DMM050079C3] Barbieri, I. and Kouzarides, T. (2020). Role of RNA modifications in cancer. *Nat. Rev. Cancer* 20, 303-322. 10.1038/S41568-020-0253-2 32300195

[DMM050079C4] Bardelli, V., Arniani, S., Pierini, V., Di Giacomo, D., Pierini, T., Gorello, P., Mecucci, C. and La Starza, R. (2021). T-cell acute lymphoblastic leukemia: Biomarkers and their clinical usefulness. *Genes* 12, 1118. 10.3390/genes12081118 34440292 PMC8394887

[DMM050079C5] Baugh, L. R. and Sternberg, P. W. (2006). DAF-16/FOXO regulates transcription of cki-1/Cip/Kip and repression of lin-4 during C. elegans L1 arrest. *Curr. Biol.* 16, 780-785. 10.1016/J.CUB.2006.03.021 16631585

[DMM050079C6] Bellelli, R., Youds, J., Borel, V., Svendsen, J., Pavicic-Kaltenbrunner, V. and Boulton, S. J. (2020). Synthetic lethality between DNA polymerase epsilon and RTEL1 in metazoan DNA replication. *Cell Rep.* 31, 107675. 10.1016/J.CELREP.2020.107675 32460026 PMC7262601

[DMM050079C7] Berry, L. W., Westlund, B. and Schedl, T. (1997). Germ-line tumor formation caused by activation of glp-1, a Caenorhabditis elegans member of the Notch family of receptors. *Development* 124, 925-936. 10.1242/dev.124.4.925 9043073

[DMM050079C8] Bhatia, V., Barroso, S. I., Garcí­Rubio, M. L., Tumini, E., Herrera-Moyano, E. and Aguilera, A. (2014). BRCA2 prevents R-loop accumulation and associates with TREX-2 mRNA export factor PCID2. *Nature* 511, 362-365. 10.1038/NATURE13374 24896180

[DMM050079C9] Boxem, M. and Van Den Heuvel, S. (2001). *lin-35* Rb and *cki-1* Cip/Kip cooperate in developmental regulation of G1 progression in *C. elegans*. *Development* 128, 4349-4359. 10.1242/DEV.128.21.4349 11684669

[DMM050079C10] Boxem, M. and Van Den Heuvel, S. (2002). C. elegans class B synthetic multivulva genes act in G(1) regulation. *Curr. Biol.* 12, 906-911. 10.1016/S0960-9822(02)00844-8 12062054

[DMM050079C11] Brady, S. C., Zdraljevic, S., Bisaga, K. W., Tanny, R. E., Cook, D. E., Lee, D., Wang, Y. and Andersen, E. C. (2019). A novel gene underlies bleomycin-response variation in Caenorhabditis elegans. *Genetics* 212, 1453-1468. 10.1534/GENETICS.119.302286 31171655 PMC6707474

[DMM050079C12] Brantley, S. J., Cotten, S. W., Lamson, D. R., Smith, G. R., Liu, R. and Williams, K. P. (2017). Discovery of small molecule inhibitors for the C. elegans caspase CED-3 by high-throughput screening. *Biochem. Biophys. Res. Commun.* 491, 773-779. 10.1016/j.bbrc.2017.07.100 28733033 PMC5590106

[DMM050079C13] Bujarrabal-Dueso, A., Sendtner, G., Meyer, D. H., Chatzinikolaou, G., Stratigi, K., Garinis, G. A. and Schumacher, B. (2023). The DREAM complex functions as conserved master regulator of somatic DNA-repair capacities. *Nat. Struct. Mol. Biol.* 30, 475-488. 10.1038/s41594-023-00942-8 36959262 PMC10113156

[DMM050079C14] Burton, N. O., Willis, A., Fisher, K., Braukmann, F., Price, J., Stevens, L., Baugh, L. R., Reinke, A. and Miska, E. A. (2021). Intergenerational adaptations to stress are evolutionarily conserved, stress-specific, and have deleterious trade-offs. *eLife* 10, e73425. 10.7554/ELIFE.73425 34622777 PMC8570697

[DMM050079C15] Camacho, J., Truong, L., Kurt, Z., Chen, Y.-W., Morselli, M., Gutierrez, G., Pellegrini, M., Yang, X. and Allard, P. (2018). The memory of environmental chemical exposure in C. elegans is dependent on the jumonji demethylases jmjd-2 and jmjd-3/utx-1. *Cell Rep.* 23, 2392-2404. 10.1016/J.CELREP.2018.04.078 29791850 PMC6003705

[DMM050079C16] Cao, J., Packer, J. S., Ramani, V., Cusanovich, D. A., Huynh, C., Daza, R., Qiu, X., Lee, C., Furlan, S. N., Steemers, F. J. et al. (2017). Comprehensive single-cell transcriptional profiling of a multicellular organism. *Science* 357, 661-667. 10.1126/science.aam8940 28818938 PMC5894354

[DMM050079C17] Ceol, C. J., Stegmeier, F., Harrison, M. M. and Horvitz, H. R. (2006). Identification and classification of genes that act antagonistically to let-60 Ras signaling in Caenorhabditis elegans vulval development. *Genetics* 173, 709-726. 10.1534/GENETICS.106.056465 16624904 PMC1526536

[DMM050079C18] Ceron, J., Rual, J.-F., Chandra, A., Dupuy, D., Vidal, M. and Van Den Heuvel, S. (2007). Large-scale RNAi screens identify novel genes that interact with the C. elegans retinoblastoma pathway as well as splicing-related components with synMuv B activity. *BMC Dev. Biol.* 7, 30. 10.1186/1471-213X-7-30 17417969 PMC1863419

[DMM050079C19] Chen, Z. and Han, M. (2001). Role of *C. elegans lin-40* MTA in vulval fate specification and morphogenesis. *Development* 128, 4911-4921. 10.1242/DEV.128.23.4911 11731470

[DMM050079C20] Chen, J., Tang, L. Y., Powell, M. E., Jordan, J. M. and Lee, S. (2022). Genetic analysis of daf-18/PTEN missense mutants for starvation resistance and developmental regulation during Caenorhabditis elegans L1 arrest. *G3* 12, jkac092. 10.1093/G3JOURNAL/JKAC092 35451480 PMC9157142

[DMM050079C21] Clark, S. G., Stern, M. J. and Horvritz, H. R. (1992). C. elegans cell-signalling gene sem-5 encodes a protein with SH2 and SH3 domains. *Nature* 356, 340-344. 10.1038/356340A0 1372395

[DMM050079C22] Clucas, C., Cabello, J., Büssing, I., Schnabel, R. and Johnstone, I. L. (2002). Oncogenic potential of a C.elegans cdc25 gene is demonstrated by a gain-of-function allele. *EMBO J.* 21, 665-674. 10.1093/EMBOJ/21.4.665 11847114 PMC125848

[DMM050079C23] Conradt, B., Wu, Y. C. and Xue, D. (2016). Programmed cell death during Caenorhabditis elegans development. *Genetics* 203, 1533-1562. 10.1534/genetics.115.186247 27516615 PMC4981262

[DMM050079C24] Cook, D. E., Zdraljevic, S., Tanny, R. E., Seo, B., Riccardi, D. D., Noble, L. M., Rockman, M. V., Alkema, M. J., Braendle, C., Kammenga, J. E. et al. (2016). The genetic basis of natural variation in Caenorhabditis elegans telomere length. *Genetics* 204, 371-383. 10.1534/genetics.116.191148 27449056 PMC5012401

[DMM050079C25] Coraggio, F., Püschel, R., Marti, A. and Meister, P. (2018). Polycomb and Notch signaling regulate cell proliferation potential during Caenorhabditis elegans life cycle. *Life Sci. Alliance* 2, e201800170. 10.26508/LSA.201800170 30599047 PMC6306570

[DMM050079C26] Corchado-Sonera, M., Rambani, K., Navarro, K., Kladney, R., Dowdle, J., Leone, G. and Zetka, M. (2022). Discovery of nonautonomous modulators of activated Ras. *G3* 12, jkac200. 10.1093/G3JOURNAL/JKAC200 35929788 PMC9526067

[DMM050079C27] Crabbe, L., Verdun, R. E., Haggblom, C. I. and Karlseder, J. (2004). Defective telomere lagging strand synthesis in cells lacking WRN helicase activity. *Science* 306, 1951-1953. 10.1126/science.1103619 15591207

[DMM050079C28] Dalpe, G., Tarsitano, M., Persico, M. G., Zheng, H. and Culotti, J. (2013). *C. elegans* PVF-1 inhibits permissive UNC-40 signalling through CED-10 GTPase to position the male ray 1 sensillum. *Development* 140, 4020-4030. 10.1242/DEV.095190 24004945

[DMM050079C29] Davis, P., Zarowiecki, M., Arnaboldi, V., Becerra, A., Cain, S., Chan, J., Chen, W. J., Cho, J., Da Veiga Beltrame, E., Diamantakis, S. et al. (2022). WormBase in 2022-data, processes, and tools for analyzing Caenorhabditis elegans. *Genetics* 220, iyac003. 10.1093/GENETICS/IYAC003 35134929 PMC8982018

[DMM050079C30] Deberardinis, R. J. and Chandel, N. S. (2020). We need to talk about the Warburg effect. *Nat. Metab.* 2, 127-129. 10.1038/S42255-020-0172-2 32694689

[DMM050079C31] Delaney, K., Strobino, M., Wenda, J. M., Pankowski, A. and Steiner, F. A. (2019). H3.3K27M-induced chromatin changes drive ectopic replication through misregulation of the JNK pathway in C. elegans. *Nat. Commun.* 10, 2529. 10.1038/S41467-019-10404-9 31175278 PMC6555832

[DMM050079C32] Deng, T., Stempor, P., Appert, A., Daube, M., Ahringer, J., Hajnal, A. and Lattmann, E. (2020). The Caenorhabditis elegans homolog of the Evi1 proto-oncogene, egl-43, coordinates G1 cell cycle arrest with pro-invasive gene expression during anchor cell invasion. *PLoS Genet.* 16, e1008470. 10.1371/JOURNAL.PGEN.1008470 32203506 PMC7117773

[DMM050079C33] Derry, W. B., Putzke, A. P. and Rothman, J. H. (2001). Caenorhabditis elegans p53: role in apoptosis, meiosis, and stress resistance. *Science* 294, 591-595. 10.1126/SCIENCE.1065486 11557844

[DMM050079C34] Derry, W. B., Bierings, R., Van Iersel, M., Satkunendran, T., Reinke, V. and Rothman, J. H. (2007). Regulation of developmental rate and germ cell proliferation in Caenorhabditis elegans by the p53 gene network. *Cell death and differentiation*. *Cell Death Differ.* 14, 662-670. 10.1038/SJ.CDD.4402075 17186023

[DMM050079C35] Dirksen, P., Assié, A., Zimmermann, J., Zhang, F., Tietje, A.-M., Marsh, S. A., Félix, M.-A., Shapira, M., Kaleta, C., Schulenburg, H. et al. (2020). CeMbio - The Caenorhabditis elegans Microbiome Resource. *G3* 10, 3025-3039. 10.1534/G3.120.401309 32669368 PMC7466993

[DMM050079C36] Douglas, A. E. (2019). Simple animal models for microbiome research. *Nat. Rev. Microbiol.* 17, 764-775. 10.1038/s41579-019-0242-1 31417197

[DMM050079C37] Ellis, H. M. and Horvitz, H. R. (1986). Genetic control of programmed cell death in the nematode C. elegans. *Cell* 44, 817-829. 10.1016/0092-8674(86)90004-8 3955651

[DMM050079C38] Fay, D. S., Keenan, S. and Han, M. (2002). . fzr-1 and lin-35/Rb function redundantly to control cell proliferation in C. elegans as revealed by a nonbiased synthetic screen. *Genes Dev.* 16, 503-517. 10.1101/GAD.952302 11850412 PMC155341

[DMM050079C39] Fernández-Majada, V., Welz, P.-S., Ermolaeva, M. A., Schell, M., Adam, A., Dietlein, F., Komander, D., Büttner, R., Thomas, R. K., Schumacher, B. et al. (2016). The tumour suppressor CYLD regulates the p53 DNA damage response. *Nat. Commun.* 7, 12508. 10.1038/NCOMMS12508 27561390 PMC5007442

[DMM050079C40] Fire, A., Xu, S. Q., Montgomery, M. K., Kostas, S. A., Driver, S. E. and Mello, C. C. (1998). Potent and specific genetic interference by double-stranded RNA in Caenorhabditis elegans. *Nature* 391, 806-811. 10.1038/35888 9486653

[DMM050079C41] Fry, A. L., Webster, A. K., Burnett, J., Chitrakar, R., Baugh, L. R. and Hubbard, E. J. A. (2021). DAF-18/PTEN inhibits germline zygotic gene activation during primordial germ cell quiescence. *PLoS Genet.* 17, e1009650. 10.1371/journal.pgen.1009650 34288923 PMC8294487

[DMM050079C42] García-González, A. P., Ritter, A. D., Shrestha, S., Andersen, E. C., Yilmaz, L. S. and Walhout, A. J. M. (2017). bacterial metabolism affects the C. elegans Response to cancer chemotherapeutics. *Cell* 169, 431-441.e8. 10.1016/J.CELL.2017.03.046 28431244 PMC5484065

[DMM050079C43] García-Rodríguez, F. J., Martínez-Fernández, C., Brena, D., Kukhtar, D., Serrat, X., Nadal, E., Boxem, M., Honnen, S., Miranda-Vizuete, A., Villanueva, A. et al. (2018). Genetic and cellular sensitivity of Caenorhabditis elegans to the chemotherapeutic agent cisplatin. *Dis. Model. Mech.* 11, dmm033506. 10.1242/DMM.033506 29752286 PMC6031354

[DMM050079C44] Gartner, A. and Engebrecht, J. (2022). DNA repair, recombination, and damage signaling. *Genetics* 220, iyab178. 10.1093/GENETICS/IYAB178 35137093 PMC9097270

[DMM050079C45] Gartner, A., Milstein, S., Ahmed, S., Hodgkin, J. and Hengartner, M. O. (2000). A conserved checkpoint pathway mediates DNA damage--induced apoptosis and cell cycle arrest in C. elegans. *Mol. Cell* 5, 435-443. 10.1016/S1097-2765(00)80438-4 10882129

[DMM050079C46] Gharaibeh, R. Z. and Jobin, C. (2019). Microbiota and cancer immunotherapy: in search of microbial signals. *Gut* 68, 385-388. 10.1136/GUTJNL-2018-317220 30530851 PMC6580757

[DMM050079C47] Girard, L. R., Fiedler, T. J., Harris, T. W., Carvalho, F., Antoshechkin, I., Han, M., Sternberg, P. W., Stein, L. D. and Chalfie, M. (2007). WormBook: the online review of Caenorhabditis elegans biology. *Nucleic Acids Res.* 35, D472-D475. 10.1093/NAR/GKL894 17099225 PMC1669767

[DMM050079C48] Giunti, S., Andersen, N., Rayes, D. and De Rosa, M. J. (2021). Drug discovery: Insights from the invertebrate Caenorhabditis elegans. *Pharmacol. Res. Perspect.* 9, e00721. 10.1002/prp2.721 33641258 PMC7916527

[DMM050079C49] Goetsch, P. D., Garrigues, J. M. and Strome, S. (2017). Loss of the Caenorhabditis elegans pocket protein LIN-35 reveals MuvB's innate function as the repressor of DREAM target genes. *PLoS Genet.* 13, e1007088. 10.1371/JOURNAL.PGEN.1007088 29091720 PMC5683655

[DMM050079C50] Gómez-Orte, E., Cornes, E., Zheleva, A., Sáenz-Narciso, B., De Toro, M., IIguez, M. Ã.­, LñPez, R., San-Juan, J.-F., Ezcurra, B., Sacristán, B. et al. (2017). Effect of the diet type and temperature on the C. elegans transcriptome. *Oncotarget* 9, 9556-9571. 10.18632/ONCOTARGET.23563 29515753 PMC5839384

[DMM050079C51] Gonzalez, C. (2013). Drosophila melanogaster: a model and a tool to investigate malignancy and identify new therapeutics. *Nat. Rev. Cancer* 13, 172-183. 10.1038/NRC3461 23388617

[DMM050079C52] Greenberg, M. V. C. and Bourc'his, D. (2019). The diverse roles of DNA methylation in mammalian development and disease. *Nat. Rev. Mol. Cell Biol.* 20, 590-607. 10.1038/s41580-019-0159-6 31399642

[DMM050079C53] Grimbert, S., Velazquez, A. M. V. and Braendle, C. (2018). Physiological starvation promotes caenorhabditis elegans vulval induction. *G3* 8, 3069-3081. 10.1534/G3.118.200449 30037804 PMC6118308

[DMM050079C54] Gupta, B. P., Hanna-Rose, W. and Sternberg, P. W. (2012). Morphogenesis of the vulva and the vulval-uterine connection. In *WormBook**: The Online Review of C. elegans Biology [Internet]* (ed. The C. elegans Research Community), pp. 1-20. 10.1895/wormbook.1.152.1 PMC5402222 23208727

[DMM050079C55] Hanahan, D. and Monje, M. (2023). Cancer hallmarks intersect with neuroscience in the tumor microenvironment. *Cancer Cell* 41, 573-580. 10.1016/j.ccell.2023.02.012 36917953 PMC10202656

[DMM050079C200] Hanahan, D. and Weinberg, R. A. (2000). The hallmarks of cancer. *Cell* 100, 57-70. 10.1016/s0092-8674(00)81683-9 10647931

[DMM050079C201] Hanahan, D. and Weinberg, R. A. (2011). Hallmarks of cancer: the next generation. *Cell* 144, 646-674. 10.1016/j.cell.2011.02.013 21376230

[DMM050079C202] Hanahan, D. (2022). Hallmarks of cancer: new dimensions. *Cancer Discov.* 12, 31-46. 10.1158/2159-8290.CD-21-1059 35022204

[DMM050079C56] Hemmingsson, O., Kao, G., Still, M. and Naredi, P. (2010). ASNA-1 activity modulates sensitivity to cisplatin. *Cancer Res.* 70, 10321-10328. 10.1158/0008-5472.CAN-10-1548 20966125

[DMM050079C57] Horvitz, H. R. (2003). Worms, life, and death (Nobel lecture). *Chembiochem.* 4, 697-711. 10.1002/CBIC.200300614 12898619

[DMM050079C58] Houri-Zeevi, L., Teichman, G., Gingold, H. and Rechavi, O. (2021). Stress resets ancestral heritable small RNA responses. *eLife* 10, e65797. 10.7554/ELIFE.65797 33729152 PMC8021399

[DMM050079C59] Huang, S., Bjornsti, M. A. and Houghton, P. J. (2003). Rapamycins: mechanism of action and cellular resistance. *Cancer Biol. Ther.* 2, 222-232. 10.4161/cbt.2.3.360 12878853

[DMM050079C60] Hutter, H. and Suh, J. (2016). GExplore 1.4: An expanded web interface for queries on Caenorhabditis elegans protein and gene function. *Worm* 5, e1234659. 10.1080/21624054.2016.1234659 28090394 PMC5190144

[DMM050079C61] Hyun, M., Choi, S., Stevnsner, T. and Ahn, B. (2016). The Caenorhabditis elegans Werner syndrome protein participates in DNA damage checkpoint and DNA repair in response to CPT-induced double-strand breaks. *Cell. Signal.* 28, 214-223. 10.1016/j.cellsig.2015.12.006 26691982

[DMM050079C62] Ismat, A., Cheshire, A. M. and Andrew, D. J. (2013). The secreted AdamTS-A metalloprotease is required for collective cell migration. *Development* 140, 1981-1993. 10.1242/DEV.087908/-/DC1 23536567 PMC3631971

[DMM050079C63] Ji, J., Yuan, J., Guo, X., Ji, R., Quan, Q., Ding, M., Li, X. and Liu, Y. (2019). Harmine suppresses hyper-activated Ras-MAPK pathway by selectively targeting oncogenic mutated Ras/Raf in Caenorhabditis elegans. *Cancer Cell Int.* 19, 159. 10.1186/S12935-019-0880-4 31198408 PMC6558680

[DMM050079C64] Johnson, S. M., Grosshans, H., Shingara, J., Byrom, M., Jarvis, R., Cheng, A., Labourier, E., Reinert, K. L., Brown, D. and Slack, F. J. (2005). RAS is regulated by the let-7 microRNA family. *Cell* 120, 635-647. 10.1016/j.cell.2005.01.014 15766527

[DMM050079C65] Johnson, C. D., Esquela-Kerscher, A., Stefani, G., Byrom, M., Kelnar, K., Ovcharenko, D., Wilson, M., Wang, X., Shelton, J., Shingara, J. et al. (2007). The let-7 microRNA represses cell proliferation pathways in human cells. *Cancer Res.* 67, 7713-7722. 10.1158/0008-5472.CAN-07-1083 17699775

[DMM050079C66] Ke, W., Saba, J. A., Yao, C.-H., Hilzendeger, M. A., Drangowska-Way, A., Joshi, C., Mony, V. K., Benjamin, S. B., Zhang, S., Locasale, J. et al. (2020). Dietary serine-microbiota interaction enhances chemotherapeutic toxicity without altering drug conversion. *Nat. Commun.* 11, 2587. 10.1038/S41467-020-16220-W 32444616 PMC7244588

[DMM050079C67] Keith Blackwell, T., Sewell, A. K., Wu, Z. and Han, M. (2019). TOR signaling in Caenorhabditis elegans development, metabolism, and aging. *Genetics* 213, 329-360. 10.1534/GENETICS.119.302504 31594908 PMC6781902

[DMM050079C68] Kim, S., Park, D. H. and Shim, J. (2008). Thymidylate synthase and dihydropyrimidine dehydrogenase levels are associated with response to 5-fluorouracil in Caenorhabditis elegans. *Mol. Cells* 26, 344-349. 18612238

[DMM050079C69] Kim, W., Underwood, R. S., Greenwald, I. and Shaye, D. D. (2018). OrthoList 2: A New Comparative Genomic Analysis of Human and Caenorhabditis elegans Genes. *Genetics* 210, 445-461. 10.1534/GENETICS.118.301307 30120140 PMC6216590

[DMM050079C70] Kipreos, E. T. and Van Den Heuvel, S. (2019). Developmental control of the cell cycle: Insights from Caenorhabditis elegans. *Genetics* 211, 797-829. 10.1534/genetics.118.301643 30846544 PMC6404260

[DMM050079C71] Kirienko, N. V., Mani, K. and Fay, D. S. (2010). Cancer models in Caenorhabditis elegans. *Dev. Dyn.* 239, 1413-1448. 10.1002/dvdy.22247 20175192 PMC4098942

[DMM050079C72] Kniazeva, M. and Ruvkun, G. (2019). Rhizobium induces DNA damage in Caenorhabditis elegans intestinal cells. *Proc. Natl. Acad. Sci. U.S.A.* 116, 3784-3792. 10.1073/PNAS.1815656116/-/DCSUPPLEMENTAL 30808764 PMC6397575

[DMM050079C73] Kobet, R. A., Pan, X., Zhang, B., Pak, S. C., Asch, A. S. and Lee, M.-H. (2014). Caenorhabditis elegans: a model system for anti-cancer drug discovery and therapeutic target identification. *Biomol. Ther.* 22, 371. 10.4062/BIOMOLTHER.2014.084 PMC4201220 25414766

[DMM050079C74] Koopman, M., Michels, H., Dancy, B. M., Kamble, R., Mouchiroud, L., Auwerx, J., Nollen, E. A. A. and Houtkooper, R. H. (2016). A screening-based platform for the assessment of cellular respiration in Caenorhabditis elegans. *Nat. Protoc.* 11, 1798-1816. 10.1038/NPROT.2016.106 27583642 PMC5040492

[DMM050079C75] Kornfeld, K., Hom, D. B. and Horvitz, H. R. (1995). The ksr-1 gene encodes a novel protein kinase involved in Ras-mediated signaling in C. elegans. *Cell* 83, 903-913. 10.1016/0092-8674(95)90206-6 8521514

[DMM050079C76] Krabbendam, I. E., Honrath, B., Dilberger, B., Iannetti, E. F., Branicky, R. S., Meyer, T., Evers, B., Dekker, F. J., Koopman, W. J. H., Beyrath, J. et al. (2020). SK channel-mediated metabolic escape to glycolysis inhibits ferroptosis and supports stress resistance in C. elegans. *Cell Death Dis.* 11, 263. 10.1038/S41419-020-2458-4 32327637 PMC7181639

[DMM050079C77] Kramer-Drauberg, M., Liu, J.-L., Desjardins, D., Wang, Y., Branicky, R. and Hekimi, S. (2020). ROS regulation of RAS and vulva development in Caenorhabditis elegans. *PLoS Genet.* 16, 1-26. 10.1371/journal.pgen.1008838 PMC7319342 32544191

[DMM050079C78] Krisher, R. L. and Prather, R. S. (2012). A role for the Warburg effect in preimplantation embryo development: Metabolic modification to support rapid cell proliferation. *Mol. Reprod. Dev.* 79, 311-320. 10.1002/mrd.22037 22431437 PMC3328638

[DMM050079C79] Kukhtar, D., Rubio-Peña, K., Serrat, X. and Cerón, J. (2020). Mimicking of splicing-related retinitis pigmentosa mutations in C. elegans allow drug screens and identification of disease modifiers. *Hum. Mol. Genet.* 29, 756-765. 10.1093/hmg/ddz315 31919495

[DMM050079C80] Kumar, R. and Wang, R. A. (2016). Structure, expression and functions of MTA genes. *Gene* 582, 112-121. 10.1016/j.gene.2016.02.012 26869315 PMC4785049

[DMM050079C81] Kyriakakis, E., Markaki, M. and Tavernarakis, N. (2015). Caenorhabditis elegans as a model for cancer research. *Mol. Cell. Oncol.* 2, e975027. 10.4161/23723556.2014.975027 27308424 PMC4905018

[DMM050079C82] Le, T. T. and Oudin, M. J. (2023). Understanding and modeling nerve-cancer interactions. *Dis. Model. Mech.* 16, dmm049729. 10.1242/dmm.049729 36621886 PMC9844229

[DMM050079C83] Lee, M. H. and Yoon, D. S. (2017). A phenotype-based RNAi screening for Ras-ERK/MAPK signaling-associated stem cell regulators in C. elegans. *Methods Mol. Biol.* 1622, 207-221. 10.1007/978-1-4939-7108-4_15 28674811 PMC5581955

[DMM050079C84] Li, R., Dinh, M., Maddox, P. and Ahmed, S. (2020). Direct full-length RNA sequencing reveals unexpected transcriptome complexity during Caenorhabditis elegans development. *Genome Res.* 30, 287-298. 10.1101/GR.251512.119/-/DC1 32024662 PMC7050527

[DMM050079C85] Lister-Shimauchi, E. H., Dinh, M., Maddox, P. and Ahmed, S. (2021). Gametes deficient for Pot1 telomere binding proteins alter levels of telomeric foci for multiple generations. *Commun. Biol.* 4, 158. 10.1038/S42003-020-01624-7 33542458 PMC7862594

[DMM050079C86] Lu, X. and Horvitz, H. R. (1998). . lin-35 and lin-53, two genes that antagonize a C. elegans Ras pathway, encode proteins similar to Rb and its binding protein RbAp48. *Cell* 95, 981-991. 10.1016/S0092-8674(00)81722-5 9875852

[DMM050079C87] Lu, F. M., Eliceiri, K. W., Stewart, J. and Jungck, J. (2007). WormClassroom.org: an inquiry-rich educational web portal for research resources of Caenorhabditis elegans. *CBE Life Sci. Educ.* 6, 98-108. 10.1187/CBE.06-07-0176 17548872 PMC1885908

[DMM050079C88] Lukácsi, S., Farkas, Z., Saskői, É., Bajtay, Z. and Takács-Vellai, K. (2021). Conserved and distinct elements of phagocytosis in human and C. elegans. *Int. J. Mol. Sci.* 22, 8934. 10.3390/IJMS22168934 34445642 PMC8396242

[DMM050079C89] Luth, E. S., Hodul, M., Rennich, B. J., Riccio, C., Hofer, J., Markoja, K. and Juo, P. (2021). VER/VEGF receptors regulate AMPA receptor surface levels and glutamatergic behavior. *PLoS Genet.* 17, e1009375. 10.1371/JOURNAL.PGEN.1009375 33561120 PMC7899335

[DMM050079C90] Luz, A. T., Godebo, T. R., Bhatt, D. P., Ilkayeva, O. R., Maurer, L. L., Hirschey, M. D. and Meyer, J. N. (2016). Arsenite uncouples mitochondrial respiration and induces a warburg-like effect in Caenorhabditis elegans. *Toxicol. Sci.* 154, 349-362. 10.1093/TOXSCI/KFW185 PMC4960910 27208080

[DMM050079C91] Malaiwong, N., Porta-De-La-Riva, M. and Krieg, M. (2023). FLInt: single shot safe harbor transgene integration via fluorescent landmark interference. *G3* 13, jkad041. 10.1093/g3journal/jkad041 36805659 PMC10151404

[DMM050079C92] Marquevielle, J., De Rache, A., Vialet, B., Morvan, E., Mergny, J.-L. and Amrane, S. (2022). G-quadruplex structure of the C. elegans telomeric repeat: a two tetrads basket type conformation stabilized by a non-canonical C-T base-pair. *Nucleic Acids Res.* 50, 7134-7146. 10.1093/NAR/GKAC523 35736226 PMC9262591

[DMM050079C93] Martínez-Fernández, C., Bergamino, M., Schiavi, A., Brena, D., Ventura, N., Honnen, S., Villanueva, A., Nadal, E. and Cerón, J. (2022). Insights into cisplatin-induced neurotoxicity and mitochondrial dysfunction in Caenorhabditis elegans. *Dis. Model. Mech.* 15, dmm049161. 10.1242/DMM.049161 35107130 PMC8995082

[DMM050079C94] Martínez-Jiménez, F., Muiños, F., Sentís, I., Deu-Pons, J., Reyes-Salazar, I., Arnedo-Pac, C., Mularoni, L., Pich, O., Bonet, J., Kranas, H. et al. (2020). A compendium of mutational cancer driver genes. *Nat. Rev. Cancer* 20, 555-572. 10.1038/s41568-020-0290-x 32778778

[DMM050079C95] Matus, D. Q., Lohmer, L. L., Kelley, L. C., Schindler, A. J., Kohrman, A. Q., Barkoulas, M., Zhang, W., Chi, Q. and Sherwood, D. R. (2015). Invasive cell fate requires G1 cell-cycle arrest and histone deacetylase-mediated changes in gene expression. *Dev. Cell* 35, 162-174. 10.1016/J.DEVCEL.2015.10.002 26506306 PMC4732529

[DMM050079C96] Maxeiner, S., Grolleman, J., Schmid, T., Kammenga, J. and Hajnal, A. (2019). The hypoxia-response pathway modulates RAS/MAPK-mediated cell fate decisions in Caenorhabditis elegans. *Life Sci. Alliance* 2, e201800255. 10.26508/lsa.201800255 31126994 PMC6536719

[DMM050079C97] Mcdiarmid, T. A., Au, V., Loewen, A. D., Liang, J., Mizumoto, K., Moerman, D. G. and Rankin, C. H. (2018). CRISPR-Cas9 human gene replacement and phenomic characterization in Caenorhabditis elegans to understand the functional conservation of human genes and decipher variants of uncertain significance. *Dis. Model. Mech.* 11, dmm036517. 10.1242/DMM.036517 30361258 PMC6307914

[DMM050079C98] Mclellan, J. L., O'neil, N. J., Barrett, I., Ferree, E., Van Pel, D. M., Ushey, K., Sipahimalani, P., Bryan, J., Rose, A. M. and Hieter, P. (2012). Synthetic lethality of cohesins with PARPs and replication fork mediators. *PLoS Genet.* 8, e1002574. 10.1371/JOURNAL.PGEN.1002574 22412391 PMC3297586

[DMM050079C99] Medwig-Kinney, T. N., Smith, J. J., Palmisano, N. J., Tank, S., Zhang, W. and Matus, D. Q. (2020). A developmental gene regulatory network for C. elegans anchor cell invasion. *Development* 147, dev185850. 10.1242/DEV.185850 31806663 PMC6983719

[DMM050079C100] Meier, B., clejan, I., Liu, Y., Lowden, M., Gartner, A., Hodgkin, J. and Ahmed, S. (2006). . trt-1 is the Caenorhabditis elegans catalytic subunit of telomerase. *PLoS Genet.* 2, 187-197. 10.1371/JOURNAL.PGEN.0020018 PMC1361356 16477310

[DMM050079C101] Meier, B., Volkova, N. V., Hong, Y., Bertolini, S., Gonzã¡Lez-Huici, V. Ã.­, Petrova, T., Boulton, S., Campbell, P. J., Gerstung, M. and Gartner, A. (2021). Protection of the C. elegans germ cell genome depends on diverse DNA repair pathways during normal proliferation. *PLoS ONE* 16, e0250291. 10.1371/JOURNAL.PONE.0250291 33905417 PMC8078821

[DMM050079C102] Mittal, P. and Roberts, C. W. M. (2020). The SWI/SNF complex in cancer - biology, biomarkers and therapy. *Nat. Rev. Clin. Oncol.* 17, 435-448. 10.1038/S41571-020-0357-3 32303701 PMC8723792

[DMM050079C103] Moser, S. C., Von Elsner, S., Büssing, I., Alpi, A., Schnabel, R. and Gartner, A. (2009). Functional dissection of Caenorhabditis elegans CLK-2/TEL2 cell cycle defects during embryogenesis and germline development. *PLoS Genet.* 5, e1000451. 10.1371/JOURNAL.PGEN.1000451 19360121 PMC2660272

[DMM050079C104] Mouridi, S. E., Alkhaldi, F. and Frokjar-Jensen, C. (2022). Modular safe-harbor transgene insertion for targeted single-copy and extrachromosomal array integration in Caenorhabditis elegans. *G3* 12, jkac184. 10.1093/G3JOURNAL/JKAC184 35900171 PMC9434227

[DMM050079C105] Moy, T. I., Ball, A. R., Anklesaria, Z., Casadei, G., Lewis, K. and Ausubel, F. M. (2006). Identification of novel antimicrobials using a live-animal infection model. *Proc. Natl. Acad. Sci. USA* 103, 10414-10419. 10.1073/PNAS.0604055103 16801562 PMC1482800

[DMM050079C106] Mueller, M. M., Castells-Roca, L., Babu, V., Ermolaeva, M. A., Müller, R.-U., Frommolt, P., Williams, A. B., Greiss, S., Schneider, J. I., Benzing, T. et al. (2014). DAF-16/FOXO and EGL-27/GATA promote developmental growth in response to persistent somatic DNA damage. *Nat. Cell Biol.* 16, 1168-1179. 10.1038/ncb3071 25419847 PMC4250074

[DMM050079C107] Nakdimon, I., Walser, M., Fröhli, E. and Hajnal, A. (2012). PTEN negatively regulates MAPK signaling during Caenorhabditis elegans vulval development. *PLoS Genet.* 8, e1002881. 10.1371/JOURNAL.PGEN.1002881 22916028 PMC3420937

[DMM050079C108] Nance, J. and Frøkjær-Jensen, C. (2019). The Caenorhabditis elegans transgenic toolbox. *Genetics* 212, 959-990. 10.1534/genetics.119.301506 31405997 PMC6707460

[DMM050079C109] Nguyen, T. T. M., Mai, V.-H., Kim, H. S., Kim, D., Seo, M., An, Y. J. and Park, S. (2022). Real-time monitoring of host-gut microbial interspecies interaction in anticancer drug metabolism. *J. Am. Chem. Soc.* 144, 8529-8535. 10.1021/JACS.1C10998 35535499

[DMM050079C110] O'Brown, Z. K., Boulias, K., Wang, J., Wang, S. Y., O'Brown, N. M., Hao, Z., Shibuya, H., Fady, P.-E., Shi, Y., He, C. et al. (2019). Sources of artifact in measurements of 6mA and 4mC abundance in eukaryotic genomic DNA. *BMC Genomics* 20, 445. 10.1186/S12864-019-5754-6 31159718 PMC6547475

[DMM050079C111] Özdemir, I. and Steiner, F. A. (2022). Transmission of chromatin states across generations in C. elegans. *Semin. Cell Dev. Biol.* 127, 133-141. 10.1016/J.SEMCDB.2021.11.008 34823984

[DMM050079C112] Padeken, J., Folkmann, A., Rasoloson, D. and Seydoux, G. (2019). Synergistic lethality between BRCA1 and H3K9me2 loss reflects satellite derepression. *Genes Dev.* 33, 436-451. 10.1101/GAD.322495.118/-/DC1 30804228 PMC6446544

[DMM050079C113] Paix, A., Folkmann, A., Rasoloson, D. and Seydoux, G. (2015). High efficiency, homology-directed genome editing in Caenorhabditis elegans using CRISPR-Cas9 ribonucleoprotein complexes. *Genetics* 201, 47-54. 10.1534/GENETICS.115.179382 26187122 PMC4566275

[DMM050079C114] Patel, T. and Hobert, O. (2017). Coordinated control of terminal differentiation and restriction of cellular plasticity. *eLife* 6, e24100. 10.7554/eLife.24100.001 28422646 PMC5397285

[DMM050079C115] Peña-Ramos, O. and Zhou, Z. (2022). Monitoring the recruitment and fusion of autophagosomes to phagosomes during the clearance of apoptotic cells in the nematode Caenorhabditis elegans. *Bio. Protocol.* 12, e4554. 10.21769/BioProtoc.4554 PMC9724009 36532685

[DMM050079C116] Perri, F., Pisconti, S. and Vittoria Scarpati, G. D. (2016). P53 mutations and cancer: a tight linkage. *Ann. Transl. Med.* 4, 522. 10.21037/ATM.2016.12.40 28149884 PMC5233470

[DMM050079C117] Pir, M. S., Bilgin, H. I., Sayici, A., Coşkun, F., Torun, F. M., Zhao, P., Kang, Y., Cevik, S. and Kaplan, O. I. (2022). ConVarT: a search engine for matching human genetic variants with variants from non-human species. *Nucleic Acids Res.* 50, D1172-D1178. 10.1093/NAR/GKAB939 34718716 PMC8728286

[DMM050079C118] Piulats, J. M., Vidal, A., García-Rodríguez, F. J., Muñoz, C., Nadal, M., Moutinho, C., Martínez-Iniesta, M., Mora, J., Figueras, A., Guinó, E. et al. (2018). Orthoxenografts of testicular germ cell tumors demonstrate genomic changes associated with cisplatin resistance and identify PDMP as a resensitizing agent. *Clin. Cancer Res.* 24, 3755-3766. 10.1158/1078-0432.CCR-17-1898 29618620

[DMM050079C119] Ploumi, C., Kyriakakis, E. and Tavernarakis, N. (2023). Coupling of autophagy and the mitochondrial intrinsic apoptosis pathway modulates proteostasis and ageing in Caenorhabditis elegans. *Cell Death Dis.* 14, 110. 10.1038/s41419-023-05638-x 36774344 PMC9922313

[DMM050079C120] Porta-De-La-Riva, M., Fontrodona, L., Villanueva, A. and Cerón, J. (2012). Basic *Caenorhabditis elegans* methods: synchronization and observation. *J. Vis. Exp.* e4019. 10.3791/4019 22710399 PMC3607348

[DMM050079C121] Reiner, D. J., González-Pérez, V., Der, C. J. and Cox, A. D. (2008). Use of *Caenorhabditis elegans* to evaluate inhibitors of Ras function *in vivo*. *Methods Enzymol.* 439, 425-449. 10.1016/S0076-6879(07)00430-2 18374181

[DMM050079C122] Robida-Stubbs, S., Glover-Cutter, K., Lamming, D. W., Mizunuma, M., Narasimhan, S. D., Neumann-Haefelin, E., Sabatini, D. M. and Blackwell, T. K. (2012). TOR signaling and rapamycin influence longevity by regulating SKN-1/Nrf and DAF-16/FoxO. *Cell Metab.* 15, 713-724. 10.1016/J.CMET.2012.04.007 22560223 PMC3348514

[DMM050079C123] Rubio-Peña, K., Fontrodona, L., Aristizábal-Corrales, D., Torres, S., Cornes, E., García-Rodríguez, F. J., Serrat, X., González-Knowles, D., Foissac, S., Porta-De-La-Riva, M. et al. (2015). Modeling of autosomal-dominant retinitis pigmentosa in Caenorhabditis elegans uncovers a nexus between global impaired functioning of certain splicing factors and cell type-specific apoptosis. *RNA* 21, 2119-2131. 10.1261/RNA.053397.115 26490224 PMC4647465

[DMM050079C124] Ruijtenberg, S. and Van Den Heuvel, S. (2015). G1/S inhibitors and the SWI/SNF complex control cell-cycle exit during muscle differentiation. *Cell* 162, 300-313. 10.1016/j.cell.2015.06.013 26144318

[DMM050079C125] Rutkowski, R., Hofmann, K. and Gartner, A. (2010). Phylogeny and function of the invertebrate p53 superfamily. *Cold Spring Harb. Perspect. Biol.* 2, a001131. 10.1101/CSHPERSPECT.A001131 20595397 PMC2890203

[DMM050079C126] Ryu, J. S. and Koo, H. S. (2017). The Caenorhabditis elegans WRN helicase promotes double-strand DNA break repair by mediating end resection and checkpoint activation. *FEBS Lett.* 591, 2155-2166. 10.1002/1873-3468.12724 28640365

[DMM050079C127] Saito, R. M. and Van Den Heuvel, S. (2002). Malignant worms: What cancer research can learn from C. elegans. *Cancer Investig.* 20, 264-275. 10.1081/CNV-120001153 11901546

[DMM050079C128] Saskői, É., Hujber, Z., Nyírő, G., Likó, I., Mátyási, B., Petővári, G., Mészáros, K., Kovács, A. L., Patthy, L., Supekar, S. et al. (2020). The SDHB Arg230His mutation causing familial paraganglioma alters glycolysis in a new *Caenorhabditis elegans* model. *Dis. Model. Mech.* 13, dmm044925. 10.1242/DMM.044925 32859697 PMC7578352

[DMM050079C129] Schmid, T., Snoek, L. B., Fröhli, E., Van Der Bent, M. L., Kammenga, J. and Hajnal, A. (2015). Systemic regulation of RAS/MAPK signaling by the serotonin metabolite 5-HIAA. *PLoS Genet.* 11, e1005236. 10.1371/JOURNAL.PGEN.1005236 25978500 PMC4433219

[DMM050079C130] Schroeder, N. E. and Hall, D. H. (2021). Announcement of WormAtlas partnership with the Journal of Nematology. *J. Nematol.* 53, e2021-e2090. 10.21307/JOFNEM-2021-090 PMC8571904 34761227

[DMM050079C131] Schumacher, B., Hofmann, K., Boulton, S. and Gartner, A. (2001). The C. elegans homolog of the p53 tumor suppressor is required for DNA damage-induced apoptosis. *Curr. Biol.* 11, 1722-1727. 10.1016/S0960-9822(01)00534-6 11696333

[DMM050079C132] Schwartz, M. L., Davis, M. W., Rich, M. S. and Jorgensen, E. M. (2021). High-efficiency CRISPR gene editing in C. elegans using Cas9 integrated into the genome. *PLoS Genet.* 17, e1009755. 10.1371/JOURNAL.PGEN.1009755 34748534 PMC8601624

[DMM050079C133] Schwendeman, A. R. and Shaham, S. (2016). A high-throughput small molecule screen for C. Elegans linker cell death inhibitors. *PLoS ONE* 11, e0164595. 10.1371/journal.pone.0164595 27716809 PMC5055323

[DMM050079C134] Sears, C. L. and Garrett, W. S. (2014). Microbes, microbiota, and colon cancer. *Cell Host Microbe* 15, 317-328. 10.1016/J.CHOM.2014.02.007 24629338 PMC4003880

[DMM050079C135] Seo, B. and Lee, J. (2016). Observation and quantification of telomere and repetitive sequences using fluorescence in Situ hybridization (FISH) with PNA probes in Caenorhabditis elegans. *J. Vis. Exp.* 54224. 10.3791/54224 27583462 PMC5091719

[DMM050079C136] Seo, B., Kim, C., Hills, M., Sung, S., Kim, H., Kim, E., Lim, D. S., Oh, H.-S., Choi, R. M. J., Chun, J. et al. (2015). Telomere maintenance through recruitment of internal genomic regions. *Nat. Commun.* 6, 8189. 10.1038/NCOMMS9189 26382656 PMC4595603

[DMM050079C137] Serrat, X., Kukhtar, D., Cornes, E., Esteve-Codina, A., Benlloch, H., Cecere, G. and Cerón, J. (2019). CRISPR editing of sftb-1/SF3B1 in Caenorhabditis elegans allows the identification of synthetic interactions with cancer-related mutations and the chemical inhibition of splicing. *PLoS Genet.* 15, e1008464. 10.1371/JOURNAL.PGEN.1008464 31634348 PMC6830814

[DMM050079C138] Sherwood, D. R. and Sternberg, P. W. (2003). Anchor cell invasion into the vulval epithelium in C. elegans. *Dev. Cell* 5, 21-31. 10.1016/S1534-5807(03)00168-0 12852849

[DMM050079C139] Sherwood, D. R., Butler, J. A., Kramer, J. M. and Sternberg, P. W. (2005). FOS-1 promotes basement-membrane removal during anchor-cell invasion in C. elegans. *Cell* 121, 951-962. 10.1016/J.CELL.2005.03.031 15960981

[DMM050079C140] Shtessel, L., Lowden, M. R., Cheng, C., Simon, M., Wang, K. and Ahmed, S. (2013). Caenorhabditis elegans POT-1 and POT-2 repress telomere maintenance pathways. *G3* 3, 305-313. 10.1534/g3.112.004440 23390606 PMC3564990

[DMM050079C141] Sim, S. and Hibberd, M. L. (2016). Caenorhabditis elegans susceptibility to gut Enterococcus faecalis infection is associated with fat metabolism and epithelial junction integrity. *BMC Microbiol.* 16, 6. 10.1186/s12866-016-0624-8 26769134 PMC4714453

[DMM050079C142] Singh, R., Smit, R. B., Wang, X., Wang, C., Racher, H. and Hansen, D. (2021). Reduction of Derlin activity suppresses Notchdependent tumours in the C. elegans germ line. *PLoS Genet.* 17, e1009687. 10.1371/journal.pgen.1009687 34555015 PMC8491880

[DMM050079C143] Smith, J. J., Xiao, Y., Parsan, N., Medwig-Kinney, T. N., Martinez, M. A. Q., Moore, F. E. Q., Palmisano, N. J., Kohrman, A. Q., Chandhok Delos Reyes, M., Adikes, R. C. et al. (2022). The SWI/SNF chromatin remodeling assemblies BAF and PBAF differentially regulate cell cycle exit and cellular invasion in vivo. *PLoS Genet.* 18, e1009981. 10.1371/JOURNAL.PGEN.1009981 34982771 PMC8759636

[DMM050079C144] Sondka, Z., Bamford, S., Cole, C. G., Ward, S. A., Dunham, I. and Forbes, S. A. (2018). The COSMIC Cancer Gene Census: describing genetic dysfunction across all human cancers. *Nature Reviews Cancer* 18, 696-705. 10.1038/s41568-018-0060-1 30293088 PMC6450507

[DMM050079C145] Stiernagle, T. (2006). Maintenance of C. elegans. In *WormBook* (ed. The C. elegans Research Community), pp. 1-11. 10.1895/wormbook.1.101.1 PMC4781397 18050451

[DMM050079C146] Stuelten, C. H., Parent, C. A. and Montell, D. J. (2018). Cell motility in cancer invasion and metastasis: Insights from simple model organisms. *Nat. Rev. Cancer* 18, 296-312. 10.1038/nrc.2018.15 29546880 PMC6790333

[DMM050079C147] Subramanian, A., Hall, M., Hou, H., Mufteev, M., Yu, B., Yuki, K. E., Nishimura, H., Sathaseevan, A., Lant, B., Zhai, B. et al. (2021). Alternative polyadenylation is a determinant of oncogenic Ras function. *Sci. Adv.* 7, 1-21. 10.1126/sciadv.abh0562 PMC8682989 34919436

[DMM050079C148] Sulston, J. E. and Horvitz, H. R. (1977). Post-embryonic cell lineages of the nematode, Caenorhabditis elegans. *Dev. Biol.* 56, 110-156. 10.1016/0012-1606(77)90158-0 838129

[DMM050079C149] Sulston, J. E., Schierenberg, E., White, J. G. and Thomson, J. N. (1983). The embryonic cell lineage of the nematode Caenorhabditis elegans. *Dev. Biol.* 100, 64-119. 10.1016/0012-1606(83)90201-4 6684600

[DMM050079C150] Sundaram, M. V. (2006). RTK/Ras/MAPK signaling. In *WormBook* (ed. The C. elegans Research Community), pp.1-19. 10.1895/wormbook.1.80.1

[DMM050079C151] Sundaram, M. V. (2013). Canonical RTK-Ras-ERK signaling and related alternative pathways. In *WormBook* (ed. The C. elegans Research Community), pp.1-38. 10.1895/wormbook.1.80.2 PMC3885983 23908058

[DMM050079C152] Sundaram, M. and Han, M. (1995). The C. elegans ksr-1 gene encodes a novel Raf-related kinase involved in Ras-mediated signal transduction. *Cell* 83, 889-901. 10.1016/0092-8674(95)90205-8 8521513

[DMM050079C153] Tam, A. S., Chu, J. S. C. and Rose, A. M. (2015). Genome-wide mutational signature of the chemotherapeutic agent mitomycin C in Caenorhabditis elegans. *G3* 6, 133-140. 10.1534/G3.115.021915 26564951 PMC4704711

[DMM050079C154] Tate, J. G., Bamford, S., Jubb, H. C., Sondka, Z., Beare, D. M., Bindal, N., Boutselakis, H., Cole, C. G., Creatore, C., Dawson, E. et al. (2019). COSMIC: the catalogue of somatic mutations in cancer. *Nucleic Acids Res.* 47, D941-D947. 10.1093/NAR/GKY1015 30371878 PMC6323903

[DMM050079C155] Thomas, J. H., Ceol, C. J., Schwartz, H. T. and Horvitz, H. R. (2003). New genes that interact with lin-35 Rb to negatively regulate the let-60 ras pathway in Caenorhabditis elegans. *Genetics* 164, 135-151. 10.1093/GENETICS/164.1.135 12750327 PMC1462563

[DMM050079C156] Tursun, B., Patel, T., Kratsios, P. and Hobert, O. (2011). Direct conversion of C. elegans germ cells into specific neuron types. *Science* 331, 304-308. 10.1126/SCIENCE.1199082 21148348 PMC3250927

[DMM050079C157] Van Der Hoeven, D., Truong, T. N. L., Naji, A., Thapa, S., Hancock, J. F. and Van Der Hoeven, R. (2020). Identification of EGFR and RAS inhibitors using caenorhabditis elegans. *J. Vis. Exp.* 1-10. 10.3791/61788 33074252

[DMM050079C158] Van Der Vaart, A., Godfrey, M., Portegijs, V. and Van Den Heuvel, S. (2020). Dose-dependent functions of SWI/SNF BAF in permitting and inhibiting cell proliferation in vivo. *Sci. Adv.* 6, eaay3823. 10.1126/sciadv.aay3823 32494730 PMC7250657

[DMM050079C159] Van Rijnberk, L. M., Van Der Horst, S. E. M., Van Den Heuvel, S. and Ruijtenberg, S. (2017). A dual transcriptional reporter and CDK-activity sensor marks cell cycle entry and progression in C. elegans. *PLoS ONE* 12, e0171600. 10.1371/JOURNAL.PONE.0171600 28158315 PMC5291519

[DMM050079C160] Venzon, M., Das, R., Luciano, D. J., Burnett, J., Park, H. S., Devlin, J. C., Kool, E. T., Belasco, J. G., Hubbard, E. J. A. and Cadwell, K. (2022). Microbial byproducts determine reproductive fitness of free-living and parasitic nematodes. *Cell Host Microbe* 30, 786-797.e8. 10.1016/J.CHOM.2022.03.015 35413267 PMC9187612

[DMM050079C161] Vermezovic, J., Stergiou, L., Hengartner, M. O. and D'Adda Di Fagagna, F. (2012). Differential regulation of DNA damage response activation between somatic and germline cells in Caenorhabditis elegans. *Cell Death Differ.* 19, 1847-1855. 10.1038/CDD.2012.69 22705849 PMC3469062

[DMM050079C162] Vicencio, J., Martínez-Fernández, C., Serrat, X. and Cerón, J. (2019). Efficient generation of endogenous fluorescent reporters by nested CRISPR in caenorhabditis elegans. *Genetics* 211, 1143-1154. 10.1534/genetics.119.301965 30696716 PMC6456308

[DMM050079C163] Vicencio, J., Sánchez-Bolaños, C., Moreno-Sánchez, I., Brena, D., Vejnar, C. E., Kukhtar, D., Ruiz-López, M., Cots-Ponjoan, M., Rubio, A., Melero, N. R. et al. (2022). Genome editing in animals with minimal PAM CRISPR-Cas9 enzymes. *Nat. Commun.* 13, 2601. 10.1038/S41467-022-30228-4 35552388 PMC9098488

[DMM050079C164] Walker, A. C., Bhargava, R., Vaziriyan-Sani, A. S., Pourciau, C., Donahue, E. T., Dove, A. S., Gebhardt, M. J., Ellward, G. L., Romeo, T. and Czyż, D. M. (2021). Colonization of the Caenorhabditis elegans gut with human enteric bacterial pathogens leads to proteostasis disruption that is rescued by butyrate. *PLoS Pathog.* 17, e1009510. 10.1371/JOURNAL.PPAT.1009510 33956916 PMC8101752

[DMM050079C165] Wan, Q. L., Meng, X., Wang, C., Dai, W., Luo, Z., Yin, Z., Ju, Z., Fu, X., Yang, J., Ye, Q. et al. (2022). Histone H3K4me3 modification is a transgenerational epigenetic signal for lipid metabolism in Caenorhabditis elegans. *Nat. Commun.* 13, 768. 10.1038/S41467-022-28469-4 35140229 PMC8828817

[DMM050079C166] Wang, D., Kennedy, S., Conte, D., Kim, J. K., Gabel, H. W., Kamath, R. S., Mello, C. C. and Ruvkun, G. (2005). Somatic misexpression of germline P granules and enhanced RNA interference in retinoblastoma pathway mutants. *Nature* 436, 593-597. 10.1038/NATURE04010 16049496

[DMM050079C167] Wang, J., Al-Ouran, R., Hu, Y., Kim, S.-Y., Wan, Y.-W., Wangler, M. F., Yamamoto, S., Chao, H.-T., Comjean, A., Mohr, S. E. et al. (2017). MARRVEL: integration of human and model organism genetic resources to facilitate functional annotation of the human genome. *Am. J. Hum. Genet.* 100, 843-853. 10.1016/J.AJHG.2017.04.010 28502612 PMC5670038

[DMM050079C168] Wang, W., Li, J., Tan, J., Wang, M., Yang, J., Zhang, Z.-M., Li, C., Basnakian, A. G., Tang, H.-W., Perrimon, N. et al. (2021). Endonuclease G promotes autophagy by suppressing mTOR signaling and activating the DNA damage response. *Nat. Commun.* 12, 476. 10.1038/S41467-020-20780-2 33473107 PMC7817833

[DMM050079C169] Wanior, M., Krämer, A., Knapp, S. and Joerger, A. C. (2021). Exploiting vulnerabilities of SWI/SNF chromatin remodelling complexes for cancer therapy. *Oncogene* 40, 3637-3654. 10.1038/s41388-021-01781-x 33941852 PMC8154588

[DMM050079C170] Wei, H., Lambie, E. J., Osório, D. S., Carvalho, A. X. and Conradt, B. (2020). PIG-1 MELK-dependent phosphorylation of nonmuscle myosin II promotes apoptosis through CES-1 Snail partitioning. *PLoS Genet.* 16, e1008912. 10.1371/journal.pgen.1008912 32946434 PMC7527206

[DMM050079C171] Wicky, C., Villeneuve, A. M., Lauper, N., Codourey, L., Tobler, H. and Müller, F. (1996). Telomeric repeats (TTAGGC)n are sufficient for chromosome capping function in Caenorhabditis elegans. *Proc. Natl. Acad. Sci. U.S.A.* 93, 8983-8988. 10.1073/pnas.93.17.8983 8799140 PMC38581

[DMM050079C172] Wildwater, M., Sander, N., De Vreede, G. and Van Den Heuvel, S. (2011). Cell shape and Wnt signaling redundantly control the division axis of *C. elegans* epithelial stem cells. *Development* 138, 4375-4385. 10.1242/DEV.066431 21937595

[DMM050079C173] Wong, S. Q., Kumar, A. V., Mills, J. and Lapierre, L. R. (2020). C. elegans to model autophagy-related human disorders. *Prog. Mol. Biol. Transl. Sci.* 172, 325-373. 10.1016/BS.PMBTS.2020.01.007 32620247

[DMM050079C174] Xu, X. and Kim, S. K. (2012). The GATA transcription factor egl-27 delays aging by promoting stress resistance in caenorhabditis elegans. *PLoS Genet.* 8, e1003108. 10.1371/journal.pgen.1003108 23271974 PMC3521710

[DMM050079C175] Yan, A., Culp, E., Perry, J., Lau, J. T., Macneil, L. T., Surette, M. G. and Wright, G. D. (2018). Transformation of the anticancer drug doxorubicin in the human gut microbiome. *ACS Infect Dis* 4, 68-76. 10.1021/ACSINFECDIS.7B00166 29160065

[DMM050079C176] Yang, B., Xu, X., Russell, L., Sullenberger, M. T., Yanowitz, J. L. and Maine, E. M. (2019). A DNA repair protein and histone methyltransferase interact to promote genome stability in the Caenorhabditis elegans germ line. *PLoS Genet.* 15, e1007992. 10.1371/JOURNAL.PGEN.1007992 30794539 PMC6402707

[DMM050079C177] Yang, B., Schwartz, M. and Mcjunkin, K. (2020). In vivo CRISPR screening for phenotypic targets of the mir-35-42 family in C. elegans. *Genes Dev.* 34, 1227-1238. 10.1101/GAD.339333.120/-/DC1 32820039 PMC7462058

[DMM050079C178] Yoshina, S., Suehiro, Y., Kage-N., E. and Mitani, S. (2015). Locus-specific integration of extrachromosomal transgenes in C. elegans with the CRISPR/Cas9 system. *Biochem. Biophys. Rep* 5, 70-76. 10.1016/J.BBREP.2015.11.017 28955808 PMC5600330

[DMM050079C179] Yuen, G. J. and Ausubel, F. M. (2014). Enterococcus infection biology: lessons from invertebrate host models. *J. Microbiol.* 52, 200-210. 10.1007/S12275-014-4011-6 24585051 PMC4556283

[DMM050079C180] Zhang, F., Weckhorst, J. L., Assié, A., Hosea, C., Ayoub, C. A., Khodakova, A. S., Cabrera, M. L., Vidal Vilchis, D., Félix, M.-A. and Samuel, B. S. (2021). Natural genetic variation drives microbiome selection in the Caenorhabditis elegans gut. *Curr. Biol.* 31, 2603-2618.e9. 10.1016/J.CUB.2021.04.046 34048707 PMC8222194

[DMM050079C181] Zhao, F., Severson, P., Pacheco, S., Futscher, B. W. and Klimecki, W. T. (2013). Arsenic exposure induces the Warburg effect in cultured human cells. *Toxicol. Appl. Pharmacol.* 271, 72-77. 10.1016/J.TAAP.2013.04.020 23648393 PMC3714307

[DMM050079C182] Zheleva, A., Camino, L. P., Fernández-Fernández, N., García-Rubio, M., Askjaer, P., García-Muse, T. and Aguilera, A. (2021). THSC/TREX-2 deficiency causes replication stress and genome instability in Caenorhabditis elegans. *J. Cell Sci.* 134, jcs258435. 10.1242/jcs.258435 34553761 PMC10658913

[DMM050079C183] Zheng, S. Q., Ding, A.-J., Li, G.-P., Wu, G.-S. and Luo, H.-R. (2013). Drug absorption efficiency in Caenorhbditis elegans delivered by different methods. *PLoS One* 8, e56877. 10.1371/JOURNAL.PONE.0056877 23451103 PMC3581574

