# Supplementary material for: Caenorhabditis elegans for research on cancer hallmarks
Source: Dis Model Mech. 2023 Jun 6;16(6):dmm050079. doi: 10.1242/dmm.050079 (PMC10259857; doi:10.1242/dmm.050079)
Supplement: Supplementary information [file dmm-16-050079-s1.pdf]

**Table S1. List of 407 human cancer driver genes and their corresponding orthologs in *C. elegans***

Click here to download Table S1

Disease Models & Mechanisms • Supplementary information
